# Ureic clearance granule ameliorates chronic kidney disease by reshaping microbial dysbiosis via modulating bile acid metabolism

**DOI:** 10.1186/s13020-026-01423-y

**Published:** 2026-05-29

**Authors:** Li-Min Liu, Yu-Lu Zhang, Jing-Teng Zhou, Qing-Qing Yu, Wan-Ying Zhang, Wen-Feng Wang, Shu-Dan Pang, Hua Miao, Ying-Yong Zhao

**Affiliations:** 1 School of Pharmaceutical Sciences, Zhejiang Chinese Medical University, No. 548 Binwen Road, Hangzhou, 310053 Zhejiang China; 2 School of Medicine, Northwest University, Xi’an, 710069 Shaanxi China; 3 School of Pharmaceutical Sciences, Heilongjiang University of Chinese Medicine, Harbin, 150040 Heilongjiang China; 4 School of Pharmaceutical Sciences, The First Affiliated Hospital of Zhejiang Chinese Medical University, Hangzhou, 310003 Zhejiang China; 5 State Key Laboratory of Kidney Diseases, First Medical Center of Chinese, PLA General Hospital, Beijing, 100853 China

**Keywords:** Chronic kidney disease, Uremic clearance granule, Gut microbiota, Metabolomics, Bile acid metabolism, Takeda G protein-coupled receptor 5

## Abstract

**Background:**

Chronic kidney disease (CKD) is a highly prevalent global public health problem that inevitably leads to renal failure. Although renin–angiotensin system blockers, as first-line therapy, can reduce proteinuria, they cannot prevent the progression to end-stage renal disease. Therefore, the development of new treatment strategies is urgently required. The uremic clearance granule (UCG) was widely used in patients with CKD. However, the underlying molecular mechanisms of UCG for CKD treatment remain unclear.

**Methods:**

Fecal gut microbiota and serum metabolites were analyzed using metagenomics and metabolomics, respectively. The expression of extracellular matrix components, Takeda G protein-coupled receptor 5 (TGR5), glucagon-like peptide-1 receptor (GLP-1R), and nuclear factor kappa B (NF-κB) p65 was examined by in adenine-induced CKD rats.

**Results:**

UCG improved renal function and alleviated kidney fibrosis in adenine-induced CKD rats. Mechanistically, significantly altered gut bacteria, including *Helicobacter hepaticus, Gemella hemolysans, Bacteroides ovatus, Lactococcus cremoris, Bacteroides fragilis, Alistipes finegoldii, and Eubacterium limosum*, showed strong linear correlations with serum creatinine levels in CKD rats. UCG treatment improved aberrant changes in these gut bacteria, indicating that UCG can reshape gut microbiota dysbiosis. Microbial-derived metabolites act as a bridge between gut microbiota and host. Further analysis showed that serum bile acids, including ursodeoxycholic acid (UDCA), taurodeoxycholic acid, and hyodeoxycholic acid (HDCA), were strongly correlated with serum creatinine levels in CKD rats, and these aberrant metabolites were reversed by UCG treatment. Notably, both UDCA and HDCA showed strong linear correlations with *Bacteroides ovatus*, *Lactococcus cremoris*, *Bacteroides fragilis*, and* Eubacterium limosum*, suggesting that UCG regulates microbial-derived metabolites. Moreover, UCG treatment upregulated protein expression of TGR5, GLP-1R, and downregulated NF-κB p65 protein expression in the kidney tissues of CKD rats, indicating that renoprotective effects of UCG are associated with modulation of microbial dysbiosis, regulation of bile acid metabolism and improvement of TGR5, GLP-1R, and NF-κB signaling.

**Conclusions:**

This study is the first to demonstrate that UCG ameliorates CKD and renal fibrosis by reshaping microbial dysbiosis and microbial-derived bile acid metabolism. Altered gut microbiota and metabolites may serve as biomarkers to evaluate efficacy of UCG. UCG may exert its renoprotective effects by enhancing TGR5, GLP-1R, and NF-κB p65 expression through regulating microbial dysbiosis–mediated bile acid metabolism.

**Supplementary Information:**

The online version contains supplementary material available at 10.1186/s13020-026-01423-y.

## Introduction

Chronic kidney disease (CKD) is a major global health issue affecting approximately 15–20% of the adult population [1]. Renal fibrosis, particularly tubulointerstitial fibrosis, represents a common final pathological outcome of virtually all progressive forms of CKD [2]. Ultimately, patients inevitably progress to end-stage renal disease (ESRD) and require renal replacement therapies such as hemodialysis or kidney transplantation [3]. Regardless of the initial etiology, tubulointerstitial fibrosis is characterized by the activation and proliferation of fibroblasts into myofibroblasts, excessive secretion and accumulation of extracellular matrix (ECM) components, and progressive displacement of normal renal tubules [2]. Mechanistically, accumulating evidence has demonstrated that CKD is associated with hyperactivation of profibrotic molecular pathways, including the renin–angiotensin system, inhibitor of kappa B/nuclear factor kappa B (NF-κB), and TGF-β/Smad and Wnt/β-catenin signaling pathways [4–7]. Renin–angiotensin system blockers are used as first-line therapy and rescue intervention strategies for patients with CKD [8]. These agents can effectively improve renal function and reduce proteinuria; however, they cannot completely prevent progression from CKD to ESRD. Therefore, no currently available therapies are effective in halting CKD progression, and the development of new treatment strategies is urgently required.

Emerging technological advances in metagenomics and metabolomics have enabled large-scale datasets to be generated from experimental studies and patient cohorts, facilitating the exploration of disease-associated genotype–phenotype continua. Recently, several seminal studies have reported significant depletion of *L. casei Zhang, Lactobacillus johnsonii (L. johnsonii), Bacteroides fragilis (B. fragilis), Bacteroides ovatus (B. ovatus)*, and *Clostridium scindens (C. scindens)* in patients with CKD, and supplementation with these probiotics effectively improves renal function and inhibits renal fibrosis [9–12]. Extensive evidence indicates that gut microbiota–derived metabolites act as multiple intermediates between the host and gut microbiota in various refractory diseases [13, 14]. Alterations in the gut microbiome are accompanied by dysregulation of endogenous metabolites, including lipids such as diverse bile acids and arachidonic acid catabolites, as well as amino acids and their derivatives, such as indole-3-aldehyde and indole-3-lactic acid derived from tryptophan metabolism by the gut microbiota, which contribute to renal fibrosis [15–17]. A recent study demonstrated that *B. ovatus* elevated the levels of intestinal hyodeoxycholic acid (HDCA) by increasing the abundance of HDCA-producing *C. scindens* in mice [11]. HDCA promotes glucagon-like peptide-1 (GLP-1) expression by upregulating Takeda G protein-coupled receptor 5 (TGR5) and downregulating farnesoid X receptor (FXR) expression in the gut [11]. Activation of the intrarenal GLP-1 receptor delays CKD progression and mitigates renal fibrosis [11]. Moreover, further evidence showed that HDCA ameliorated renal fibrosis by directly upregulating intrarenal TGR5 expression [11]. In addition, *L. johnsonii* is an intestinal bacterial strain capable of generating indole-3-aldehyde (IAld) via tryptophan metabolism [10]. Decreased serum IAld levels are strongly and negatively correlated with creatinine levels in adenine-induced CKD rats and patients with CKD [10]. Both in vivo and in vitro studies have demonstrated that IAld treatment attenuates renal injury by inhibiting the aryl hydrocarbon receptor signaling pathway [10]. Furthermore, significant depletion of *Faecalibacterium prausnitzii (F. prausnitzii)* has been observed in patients with CKD [18]. Supplementation with *F. prausnitzii* improved renal function, reduced inflammation and serum uremic toxin levels, reshaped gut microbial ecology, and repaired intestinal integrity in CKD mice; these effects were associated with *F. prausnitzii*–derived butyrate–mediated G protein-coupled receptor 43 signaling [18]. Conversely, the relative abundance of pathogenic bacteria such as *Eggerthella lenta (E. lenta)* and *Fusobacterium nucleatum (F. nucleatum)*, was increased in patients with ESRD [19]. Supplementation with *E. lenta* or *F. nucleatum* in 5/6 nephrectomy CKD rats increased serum uremic toxin levels and exacerbated oxidative stress, inflammation, tubulointerstitial fibrosis, glomerulosclerosis, and elevations in serum creatinine and urea levels [19]. Further analysis revealed that the relative abundance of *E. lenta* and *F. nucleatum* was positively correlated with serum uremic toxin levels [19]. Metabolomics has been widely applied to the studies of biomarker identification, disease mechanisms, and drug intervention for treatment of CKD [20–24]. These findings indicate that targeting the gut microbiota and microbial-derived metabolites is an effective therapeutic strategy for CKD.

Understanding disease mechanisms is essential for the discovery of novel therapeutic agents. Accumulated clinical evidence has demonstrated the beneficial effects of traditional Chinese medicine (TCM) on CKD [25–28]. Natural products have been used for centuries for the prevention and treatment of CKD [29–33]. Increasing evidence demonstrates that natural products, such as 5,6,7,8,3′,4′-hexamethoxyflavone, madecassoside, and neohesperidin, improve renal fibrosis by increasing the abundance of *L. johnsonii, B. fragilis*, and *B. ovatus*, respectively [34, 35]. Uremic clearance granules (UCG), approved by the State Food and Drug Administration of China, are the first Chinese herbal medicine specifically developed for CKD treatment and have been used in clinical practice in China for over two decades. Several earlier randomized, double-blind, placebo-controlled, multicenter clinical trials demonstrated that UCG safely and effectively delayed CKD progression in patients with stage 3b–4 CKD, exhibited long-term efficacy in patients with moderate-to-severe kidney dysfunction, and attenuated renal function decline [36, 37]. A recent study showed that long-term UCG use improved serum creatinine variability and reduced the risk of CKD progression by 39.8% [38]. Another study reported that UCG treatment increased the estimated glomerular filtration rate (eGFR) and decreased serum uric acid and blood urea nitrogen levels, achieving statistical significance at months 3, 6, 9, 12, and 18 in patients with CKD [39]. To date, few studies have elucidated the underlying molecular mechanisms of action of UCG in CKD treatment. Several primary investigations have demonstrated that UCG ameliorates tubulointerstitial fibrosis and high glucose–induced podocyte injury by inhibiting oxidative stress and inflammation, TGF-β1 expression, advanced glycation end-products and receptors for advanced glycation end product signaling, and tubular epithelial-to-myofibroblast transdifferentiation [40, 41]. Compared to enalapril, UCG improved renal function and tubulointerstitial fibrosis by promoting ECM degradation in rats with chronic renal failure [40]. These findings suggest that UCG is a promising alternative therapy for patients with moderate to severe renal dysfunction.

In this study, we identified significantly altered fecal bacteria associated with UCG intervention to elucidate the relationship between renal function and gut microbiota in adenine-induced CKD rats using metagenomic analysis. Second, we identified UCG intervention–related serum metabolites to clarify the association between renal function and microbial-derived metabolites in CKD rats, using untargeted metabolomics. Third, we analyzed the correlations between UCG intervention–related gut bacteria and kidney function–related metabolites to identify gut microbiota–associated metabolic alterations. Finally, we examined the intrarenal expression of Takeda G protein-coupled receptor 5 (TGR5), glucagon-like peptide-1 receptor (GLP-1R), and NF-κB p65 to elucidate the microbial-derived metabolite–mediated molecular mechanisms underlying UCG intervention. This study demonstrates that UCG treatment improves CKD and inhibits renal fibrosis, which is associated with enhanced expression of TGR5 and GLP-1R as well as inhibited expression of NF-κB p65 through regulation of gut microbiota–derived metabolites.

## Materials and methods

### Chemicals, antibodies, reagents and equipment

UCG was purchased from Guangzhou Consun Pharmaceutical Co. Ltd (Guangzhou, Guangdong, China). UCG is composed of a defined mixture of Chinese herbs, as reported previously [24]. Adenine (Lot: A8626) was purchased from Sigma-Aldrich Co. (St. Louis, MO, USA). Primary antibodies against α-smooth muscle actin (α-SMA, ab32575), collagen I (ab270993), fibronectin (ab2413), E-cadherin (ab231303), zonula occludens-1 (ZO-1, ab221547), occludin (ab216327), claudin-1 (ab307692), and TGR5 (ab72608) were purchased from Abcam Company (Cambridge, MA, USA). The primary antibody against GLP-1R (#3306), and NF-κB p65 (#13,346) was purchased from Cell Signaling Technology (Danvers, MA, USA). Glyceraldehyde-3-phosphate dehydrogenase (GAPDH, 60,004-1-Ig) was purchased from Proteintech Group (Wuhan, Hubei, China). Secondary antibodies, including goat anti-rabbit immunoglobulin G (ZB-2301) and goat anti-mouse immunoglobulin G (ZB-2305), were purchased from Beijing Zhongshan Golden Bridge Biotechnology Co. Ltd (Beijing, China).

### Adenine-induced CKD rat model and drug treatment

Male Sprague–Dawley rats (6–8 weeks old, weighing 200 ± 10 g) were purchased from the Animal Center of the Xi’an Jiaotong University (Xi’an, Shaanxi, China). CKD rats were established as previously described studies [42, 43]. Briefly, rats were randomly divided into a control (CTL) group, a CKD group, and four CKD treatment groups receiving low-dose UCG (UCGL, 0.97 g/kg), medium-dose UCG (UCGM, 1.94 g/kg), high-dose UCG (UCGH, 3.88 g/kg), or enalapril (ENA, 0.02 g/kg) (n = 8 per group). CKD was induced by oral gavage of adenine at a dose of 200 mg/kg/day for three weeks, based on clinically relevant dosing. The three doses of UCG (0.97, 1.94, and 3.88 g/kg/day) were administered to CKD rats by gastric gavage for three weeks, respectively. Body weight, 24-h urine, and feces were collected during the experimental period. Rats were euthanized after anesthesia with 10% urethane at week 3, and serum and kidney tissues were collected for further analyses. All animal care and experimental procedures were approved by the Ethics Committee for Animal Experiments of Northwest University (No. 202418-06) and conducted in accordance with the Helsinki Declaration.

### Clinical biochemical analyses

Serum levels of creatinine, urea, uric acid, total cholesterol (TC), triglycerides (TG), and low-density lipoprotein-cholesterol (LDL-C) were determined using an Olympus AU640 automatic analyzer.

### Histopathological analysis

Kidney tissues were stained with hematoxylin–eosin (H&E) and Masson’s trichrome method based on our previous publication [42]. Pathological analyses were performed under a light microscope.

### Immunohistochemical analysis

Immunohistochemical staining was performed as described in our previous publication [42]. Renal tissue slides were incubated with anti-α-SMA and incubated with a secondary antibody. Pathological analyses were performed under a light microscope.

### Western blot analysis

Western blotting was performed as previously described [44]. The protein expression was normalized to that of GAPDH. The bands were quantified using ImageJ software.

### Metagenomic analysis

Based on illumina novaseq™ x plus technique, fecal samples were analyzed by metagenomic sequencing as described in our previous publication [45]. The experimental procedures, including fecal DNA extraction, quantitative real-time polymerase chain reaction amplification, quantification, pooling and sequencing, metagenomic sequence processing, taxonomic and functional profiling, and diversity analysis, were performed as previously reported [45].

### Metabolomic analysis

Serum samples were analyzed using a 2.1 mm × 100 mm ACQUITY HSS T3 column (1.8 µm) with a Waters ACQUITY™ UPLC system (Waters Corporation, Milford, MA, USA) equipped with a Waters Xevo™ G2 QTof mass spectrometer (Waters MS Technologies, Manchester, UK). The metabolomic procedures, including sample preparation, metabolite separation, mass spectrometric detection, raw data processing, and metabolite identification, were performed according to previously published protocols [44, 46].

### Statistical analysis

The number of replicates was 6–7 per group for each dataset, and the results are presented as mean ± SEM. Statistical analyses were performed using SPSS and GraphPad Prism. Differences between two groups were analyzed using unpaired Student’s t-test. Comparisons among multiple groups were conducted using one-way analysis of variance (ANOVA), followed by a post hoc test. Principal component analysis (PCA), partial least squares discriminant analysis (PLS-DA), sparse partial least squares discriminant analysis (sPLS-DA), dendrogram construction, heatmap visualization, and debiased sparse partial correlation (DSPC) analyses were performed using the SIMCA-P software and MetaboAnalyst. In some analyses, *P*-values were corrected for multiple comparisons using the Benjamini–Hochberg false discovery rate (FDR). Statistical significance was set at *P* < 0.05.

## Results

### UCG improved renal function and ameliorated kidney injury in adenine-induced CKD rats

**Fig. 1:**
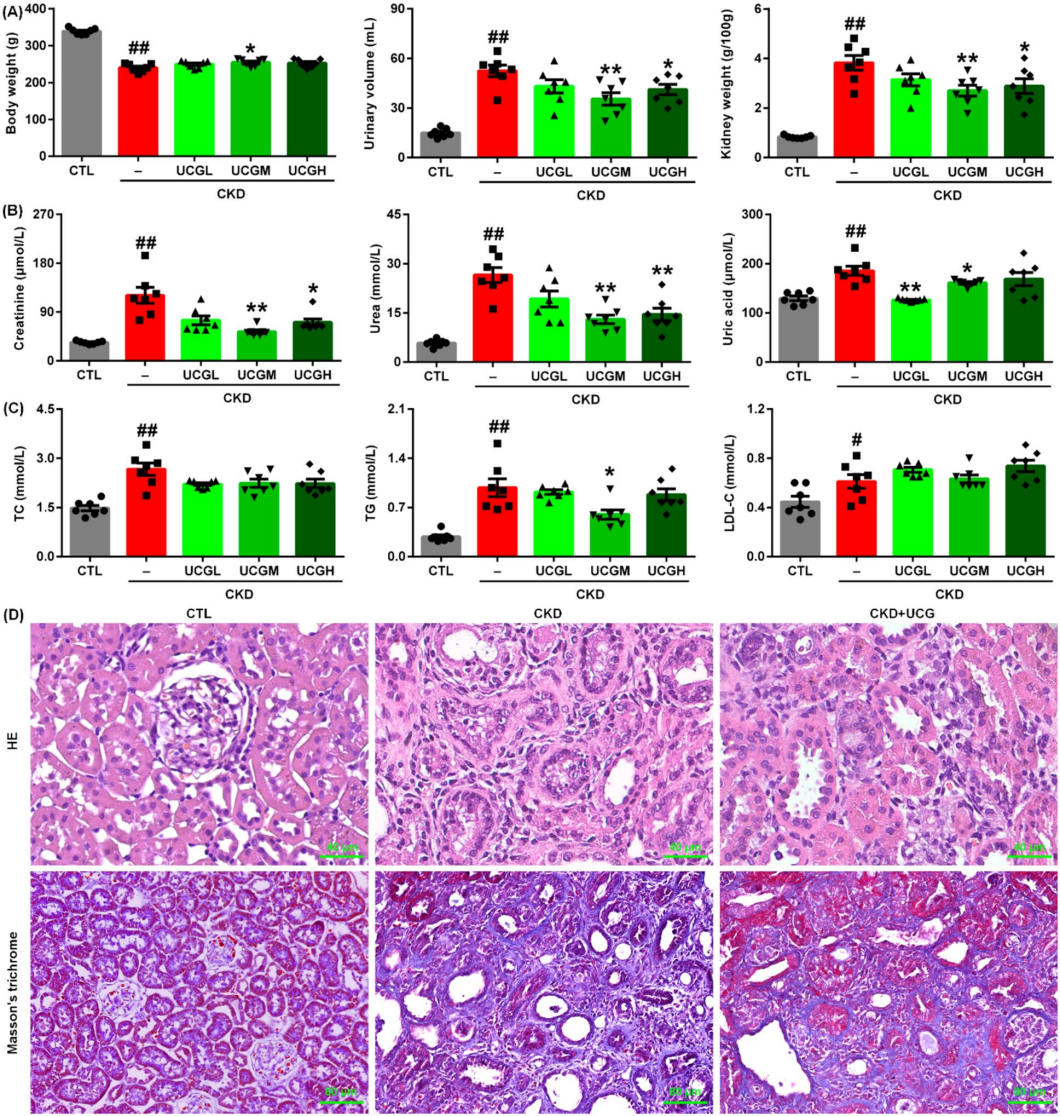
UCG improved renal function and ameliorated kidney injury in adenine-induced CKD rats. **A** Body weight, urinary volume, and kidney weight index in CTL, adenine-induced CKD, and CKD rats treated with three doses of UCG. **B** Serum levels of creatinine, urea, and uric acid in CTL, CKD, and CKD rats treated with three doses of UCG. **C** Serum levels of TC, TG, and LDL-C in CTL, adenine-induced CKD, and CKD rats treated with three doses of UCG or enalapril (ENA). **D** Representative images of H&E and Masson’s trichrome staining of kidney tissues from CTL, adenine-induced CKD, and UCG-treated CKD rats. ^#^*P* < 0.05, ^##^*P* < 0.01 compared with CTL rats; *P < 0.05, **P < 0.01 compared with CKD rats

Uric acid is the final metabolite of adenine. Oral administration of excessive exogenous adenine resulted in a significant decrease in body weight and a significant increase in urinary volume and kidney weight index in rats with adenine-induced CKD. Treatment with UCGM (1.94 g/kg) significantly increased body weight, while treatment with UCGM (1.94 g/kg) and UCGH (3.88 g/kg) significantly decreased urinary volume and kidney weight index in adenine-induced CKD rats (Fig. 1A).

Serum levels of creatinine, urea, and uric acid were significantly elevated in adenine-induced CKD rats compared to those in control rats. Treatment with UCGM (1.94 g/kg) and UCGH (3.88 g/kg) significantly reduced serum creatinine and urea levels compared with untreated CKD rats (Fig. 1B). In addition, enalapril (ENA) reduced serum creatinine levels in rats with CKD (Fig. 1B). Notably, treatment with all three doses of UCG significantly decreased serum uric acid levels in adenine-induced CKD rats compared to those in untreated CKD rats (Fig. 1B). The serum levels of TC, TG, and LDL-C were significantly higher in adenine-induced CKD rats than in control rats (Fig. 1C). However, only UCGM (1.94 g/kg) significantly reduced serum TG levels compared to untreated CKD rats (Fig. 1C). UCG at doses of 1.94 and 3.88 g/kg exhibited stronger renoprotective effects than the 0.97 g/kg dose, with 1.94 g/kg demonstrating the most pronounced efficacy. Therefore, UCG 1.94 g/kg was selected for subsequent experiments.

Histological analysis using H&E staining showed that excessive adenine administration induced severe intrarenal inflammatory cell proliferation, inflammatory infiltration, and tubulointerstitial fibrosis in rats (Fig. 1D). These pathological changes were markedly attenuated by UCG treatment (Fig. 1D). Similarly, Masson’s trichrome staining revealed severe tubulointerstitial fibrosis in adenine-induced CKD rats compared to that in control rats, which was significantly inhibited by UCG treatment (Fig. 1D). These data indicate that UCG improves renal function and inhibits renal fibrosis in rats with adenine-induced CKD.

### UCG treatment inhibited the expression of ECM components in CKD rats

**Fig. 2:**
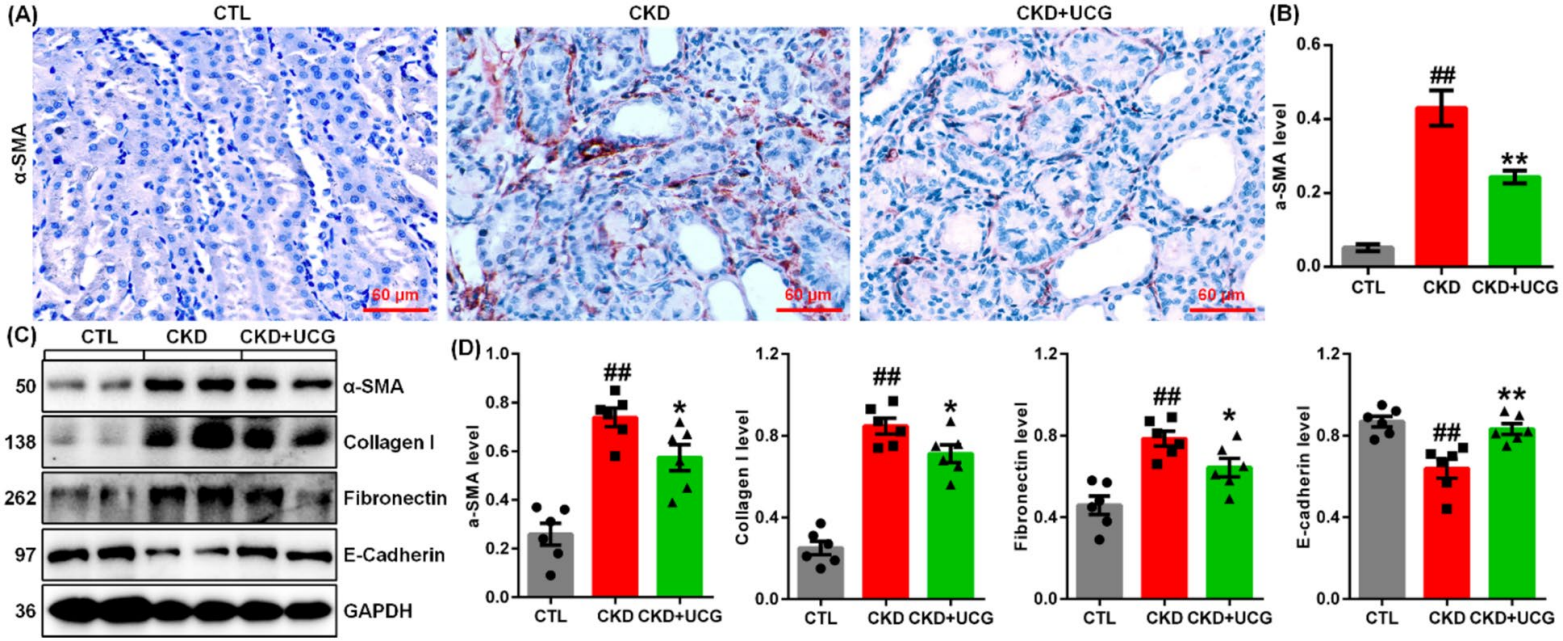
UCG treatment inhibited protein expression of ECM components in CKD rats. **A** Immunohistochemical analysis of intrarenal α-SMA expression in CTL, adenine-induced CKD, and UCG-treated CKD rats. **B** Quantitative analysis of α-SMA protein expression in the three groups. **C** Protein expression of intrarenal ECM components, including α-SMA, collagen I, and fibronectin, as well as E-cadherin, in CTL, adenine-induced CKD, and UCG-treated CKD rats. **D** Quantitative analysis of profibrotic protein expression in kidney tissues from the three groups. ^#^*P* < 0.05, ^##^*P* < 0.01 compared with CTL rats; *P < 0.05, **P < 0.01 compared with CKD rats

Immunohistochemical analysis demonstrated that excessive adenine administration markedly upregulated α-smooth muscle actin (α-SMA) expression in kidney tissues of adenine-induced CKD rats (Fig. 2A, B). UCG treatment significantly suppressed intrarenal α-SMA expressions compared to untreated CKD rats (Fig. 2A, B). Consistently, protein expression of profibrotic markers, including α-SMA, collagen I, and fibronectin, was significantly increased, whereas E-cadherin expression was decreased in the kidney tissues of CKD rats (Fig. 2C, D). UCG treatment markedly reduced the expression of these profibrotic proteins and preserved E-cadherin expression in the CKD rats (Fig. 2C, D). These findings suggest that UCG inhibits the excessive accumulation and deposition of ECM components in adenine-induced CKD rats.

### UCG treatment improved gut microbial dysbiosis in adenine-induced CKD rats

**Fig. 3:**
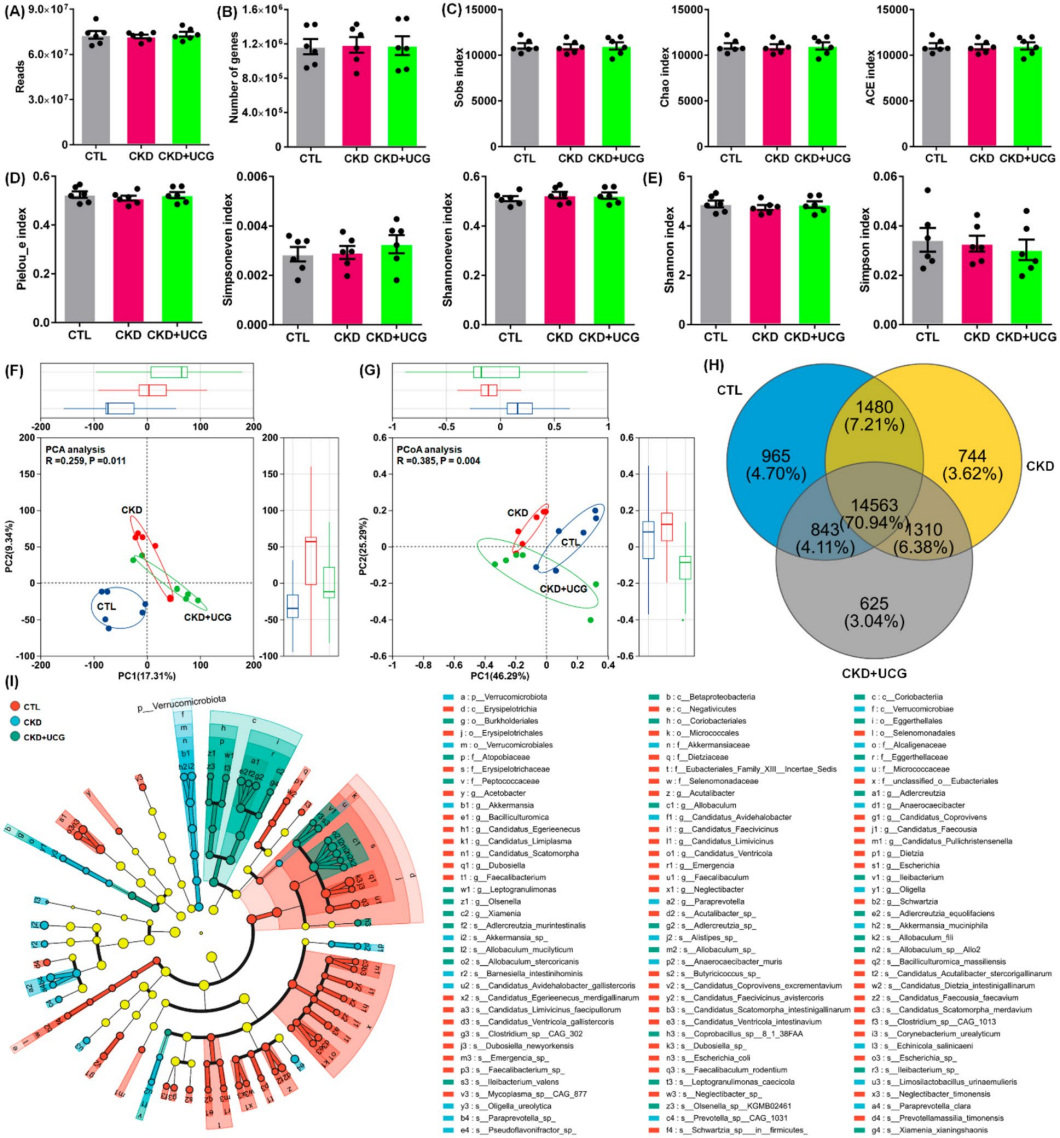
UCG treatment improved gut microbial dysbiosis in adenine-induced CKD rats. **A** Sequencing reads from fecal samples of CTL, adenine-induced CKD, and UCG-treated CKD rats. **B** Gene counts from fecal samples of the three groups. **C** Community richness indices (sobs, chao, ace) at the species level. **D** Community evenness indices (Pielou_e, simpsoneven, shannoneven) at the species level. **E** Community diversity indices (Shannon, Simpson) at the species level. **F** PCA of the three groups at the species level. **G** PCoA of the three groups at the species level. **H** Venn diagram showing the number of shared significantly altered bacteria among CTL, adenine-induced CKD, and UCG-treated CKD rats. **I** Cladogram of differentially abundant taxa based on phylum-to-species-level data (LDA threshold > 2.4)

The fecal gut microbiota of rats was analyzed using metagenomic sequencing. CKD rats showed slight changes in the read counts and gene numbers (Fig. 3A). The α-diversity indices, including community richness indices (Sobs, Chao, and Ace), community evenness indices (Pielou_e, Simpson_even, and Shannon_even), and community diversity indices (Shannon and Simpson), did not show statistically significant differences between groups at the species level (Fig. 3C–E). Both PCA and principal coordinates analysis (PCoA) demonstrated a clear separation between CKD and CTL rats (Fig. 3F, G). Notably, UCG-treated CKD rats were positioned between the CKD and CTL groups in the PCA plot (Fig. 3F). Consistently, the PCoA score plot distinguished between CTL, CKD, and UCG-treated CKD rats (Fig. 3G). Venn diagram analysis showed that 14,563 genes were shared among the CTL, CKD, and UCG-treated CKD rats at the species level (Fig. 3H). Analysis of abundant taxa from the phylum to species levels revealed distinct changes in the structure and composition of the gut microbiota in the feces of the CTL, CKD, and UCG-treated CKD rats. The results showed that most bacteria are abundant in CTL rats, but their abundance is reduced in CKD rats. UCG intervention inhibited the decrease in bacterial abundance (Fig. 3I). These findings indicate that UCG treatment improves gut microbiota dysbiosis in adenine-induced CKD rats.

### UCG treatment reshaped lipid metabolism–related gut microbial dysbiosis in CKD rats

**Fig. 4:**
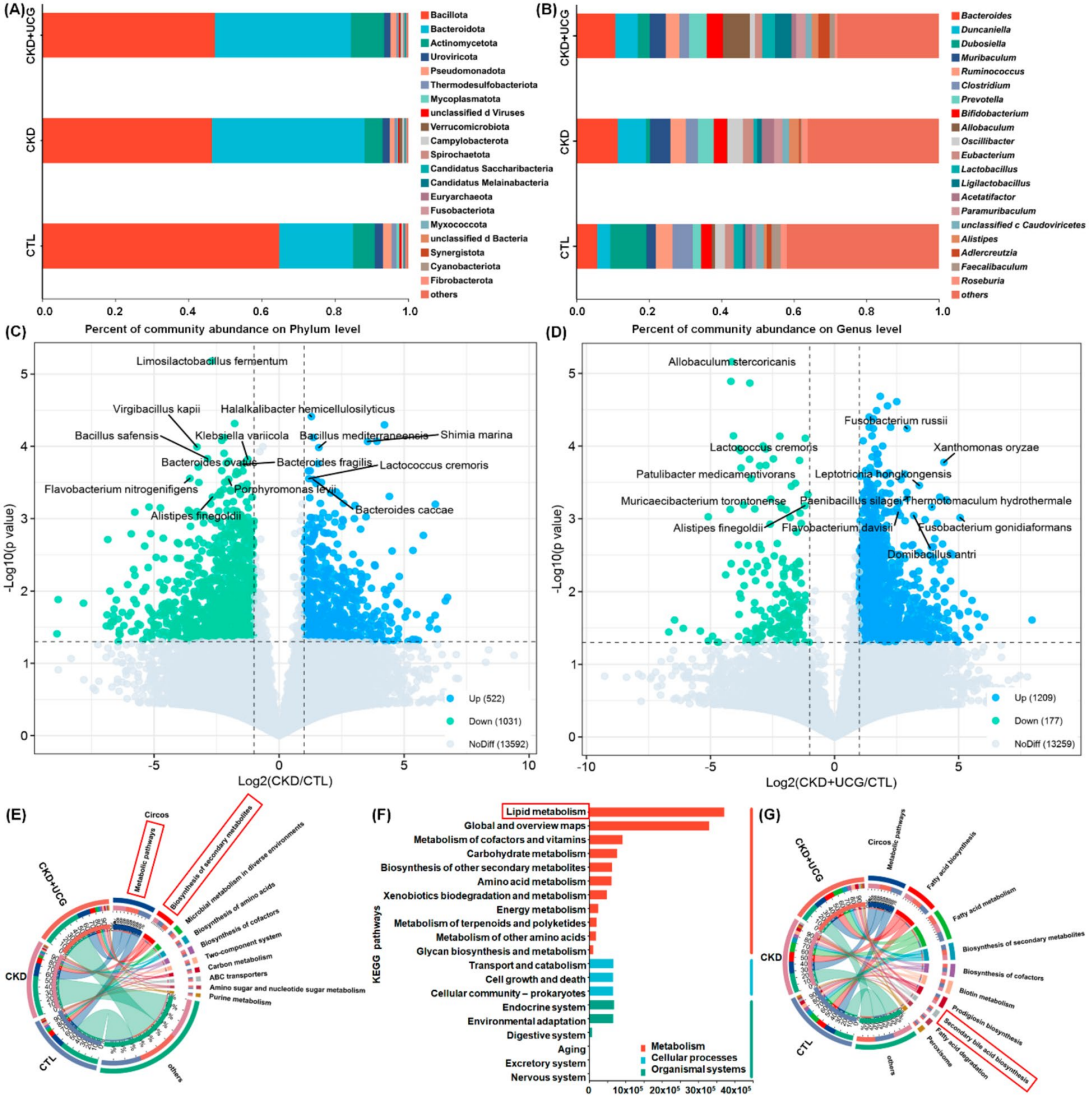
UCG treatment reshaped lipid metabolism–related gut microbial dysbiosis in CKD rats. **A** Taxonomic distribution of bacteria in CTL, adenine-induced CKD, and UCG-treated CKD rats at the phylum level (top 20). **B** Taxonomic distribution of bacteria in the three groups at the species level (top 20). **C** Volcano plot of relative abundance at the species level (CKD/CTL). **D** Volcano plot of relative abundance at the species level (CKD + UCG/CKD). Red dots indicate taxa selected with CKD + UCG/CKD > 1.0 and *P* < 0.05. **E** Circos plot of KEGG pathway analysis of bacteria across the three groups (level 2). **F** KEGG pathway analysis of bacteria across the three groups. **G** Circos plot of KEGG pathway analysis of bacteria across the three groups (level 3)

At the phylum level, all three groups were predominantly composed of Bacillota and Bacteroidetes. Compared with the CTL rats, CKD rats exhibited a significant decrease in the relative abundance of Bacillota and a significant increase in the relative abundance of Bacteroidota in the fecal samples (Fig. 4A). At the genus level, the three groups were mainly dominated by *Bacteroides*, *Duncaniella*, and *Dubosiella*. Compared with the CTL rats, CKD rats showed a significant increase in the relative abundance of *Bacteroides* and *Duncaniella* and a significant decrease in the relative abundance of *Dubosiella* in fecal samples (Fig. 4B). However, UCG treatment did not significantly affect these bacterial taxa at the phylum or genus level compared to untreated CKD rats (Fig. 4A, B). Based on fold change values and *P-values*, volcano plot analysis identified 1,553 prominent bacterial taxa in the CKD/CTL comparison, including *Shimia marina*, *Haloimpatiens lingqiaonensis*, *Desulfovirgula thermocuniculi*, *Bacillus safensis*, *B. fragilis*, and *Porphyromonas levii*, as well as 1,386 prominent bacterial taxa in the CKD + UCG/CTL comparison, including *Lactococcus cremoris* (*L. cremoris*), *Alistipes finegoldii* (*A. finegoldii*), *Paenibacillus silage*, and *Fusobacterium russii* (Fig. 4C, D). These taxa were strongly associated with lipid metabolism (Fig. 4E–G).

To explore the potential functions of the significantly altered gut microbiota, the functional composition of the bacterial metagenome was predicted using KEGG orthology analysis. The results showed that 10 major pathways, including metabolic pathways and biosynthesis of secondary metabolites, were associated with microbial dysbiosis in CKD and UCG-treated CKD rats (Fig. 4E). KEGG functional analysis further indicated that lipid metabolism was the most prominently affected pathway (Fig. 4F), suggesting that CKD and UCG treatments exert substantial effects on lipid metabolism. Further analysis of lipid metabolism pathways revealed that fatty acid biosynthesis and metabolism, biosynthesis of secondary metabolites, and secondary bile acid biosynthesis were closely associated with microbial dysbiosis in CKD and UCG-treated CKD rats (Fig. 4G). Therefore, these data suggest that lipid metabolism, particularly secondary bile acid biosynthesis, plays a critical role in CKD and UCG therapy.

### UCG treatment sculpted bile acid metabolism–related gut bacteria in CKD rats

**Fig. 5:**
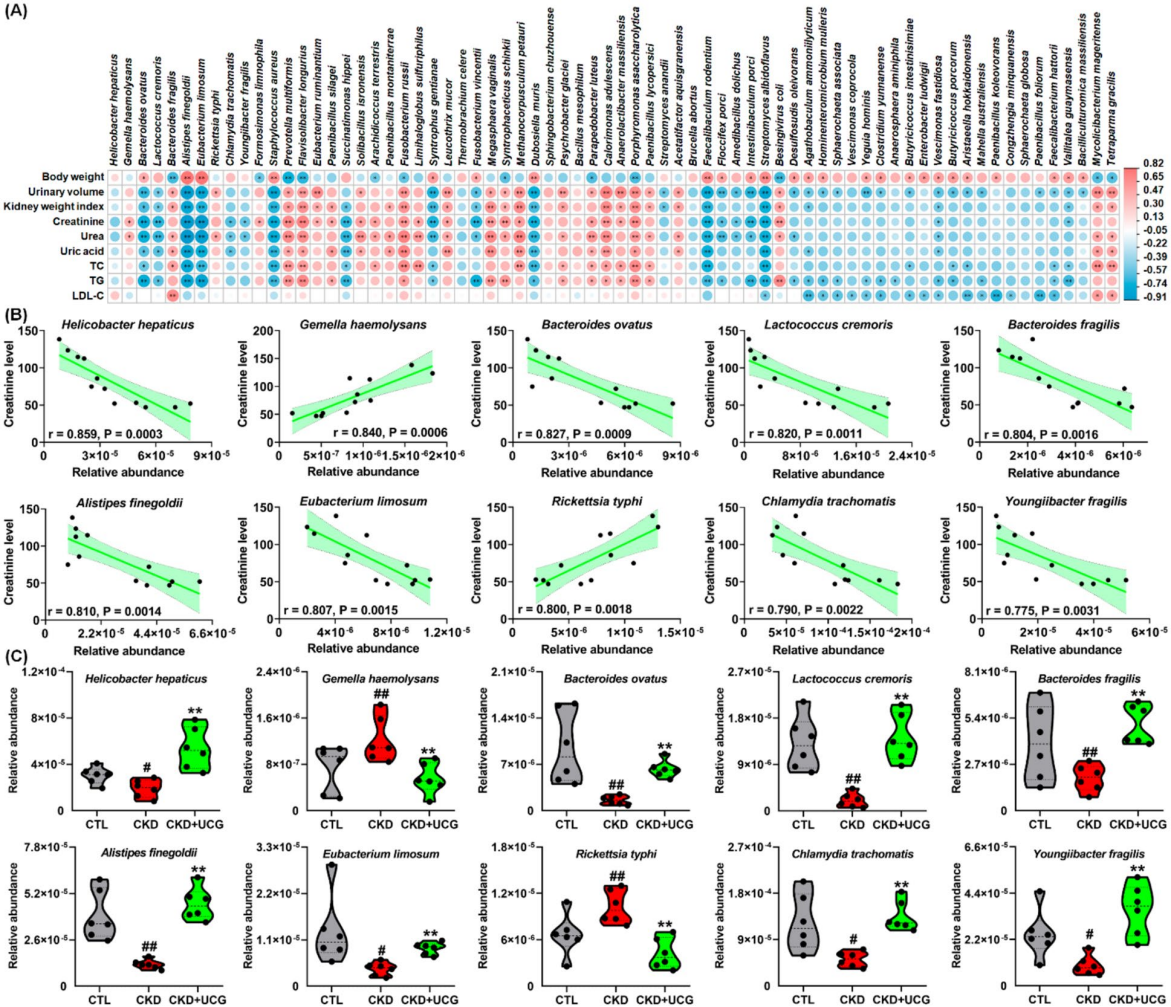
UCG treatment sculpted bile acid metabolism–associated gut bacteria in CKD rats. **A** Correlation heatmap of 70 significantly altered bacteria (CKD vs. CTL and CKD + UCG vs. CKD; *P* < 0.05) and nine clinical indices based on Spearman correlation coefficients. Circle size indicates correlation strength; red indicates positive correlation and blue indicates negative correlation. **B** Linear correlations between serum creatinine levels and relative abundance of 10 significantly altered bacteria (*P* < 0.05). Red shading indicates 95% confidence bands. **C** Relative abundance of 10 significantly altered bacteria in CTL, adenine-induced CKD, and UCG-treated CKD rats. ^#^*P* < 0.05, ^##^*P* < 0.01 compared with CTL rats; *P < 0.05, **P < 0.01 compared with CKD rats

Based on P < 0.05 for both CKD versus CTL and CKD + UCG versus CKD comparisons, 70 altered bacterial taxa were selected (Fig. 5A). To determine whether these 70 bacteria were associated with kidney function, Spearman’s correlation analysis was performed between their relative abundances and nine clinical parameters, including body weight, urinary volume, kidney weight index, creatinine, urea, uric acid, TC, TG, and LDL-C. As shown in Fig. 5A, the relative abundances of *B. ovatus, A. finegoldii*, and *Eubacterium limosum (E. limosum)* were positively correlated with body weight and negatively correlated with urinary volume, kidney weight index, creatinine, urea, uric acid, TC, and TG levels. These correlations suggest that significantly altered gut bacteria are associated with adenine-induced renal injury.

Creatinine is an important biomarker for evaluating kidney function and diagnosing CKD. To further investigate the relationship between significantly altered bacteria and renal function, linear regression analysis was conducted between the relative abundances of the 70 bacteria and serum creatinine levels in the CKD and UCG-treated CKD rats. Among these taxa, several bacteria, particularly *Helicobacter hepaticus (H. hepaticus), Gemella haemolysans (G. haemolysans), Bacteroides ovatus, Lactococcus cremoris (L. cremoris), Bacteroides fragilis, Anaerostipes finegoldii*, and *Enterococcus limosum*, showed strong correlations with serum creatinine levels, with correlation coefficients greater than 0.80 (Fig. 5B). These results indicate that these bacteria may serve as potential biomarkers for predicting the therapeutic effects of UCG consumption.

Oral administration of excessive adenine resulted in a significant decrease in the relative abundances of *H. hepaticus, B. ovatus, L. cremoris, B. fragilis, A. finegoldii, E. limosum, Chlamydia trachomatis*, and *Youngiibacter fragilis*, along with a significant increase in the relative abundances of *G. haemolysans* and *Rickettsia typhi* in the feces of CKD rats compared with CTL rats (Fig. 5C). Notably, UCG treatment reversed these aberrant bacterial changes in the CKD rats (Fig. 5C). Several recent seminal studies have demonstrated that *H. hepaticus, B. ovatus, B. fragilis*, and *E. limosum* are closely associated with bile acid production and metabolism [11, 12, 47, 48]. These findings suggest that UCG treatment may influence bile acid metabolism–related gut bacteria in CKD rats.

### UCG antifibrotic effects were associated with altered bile acid metabolic profiles in CKD rats

**Fig. 6:**
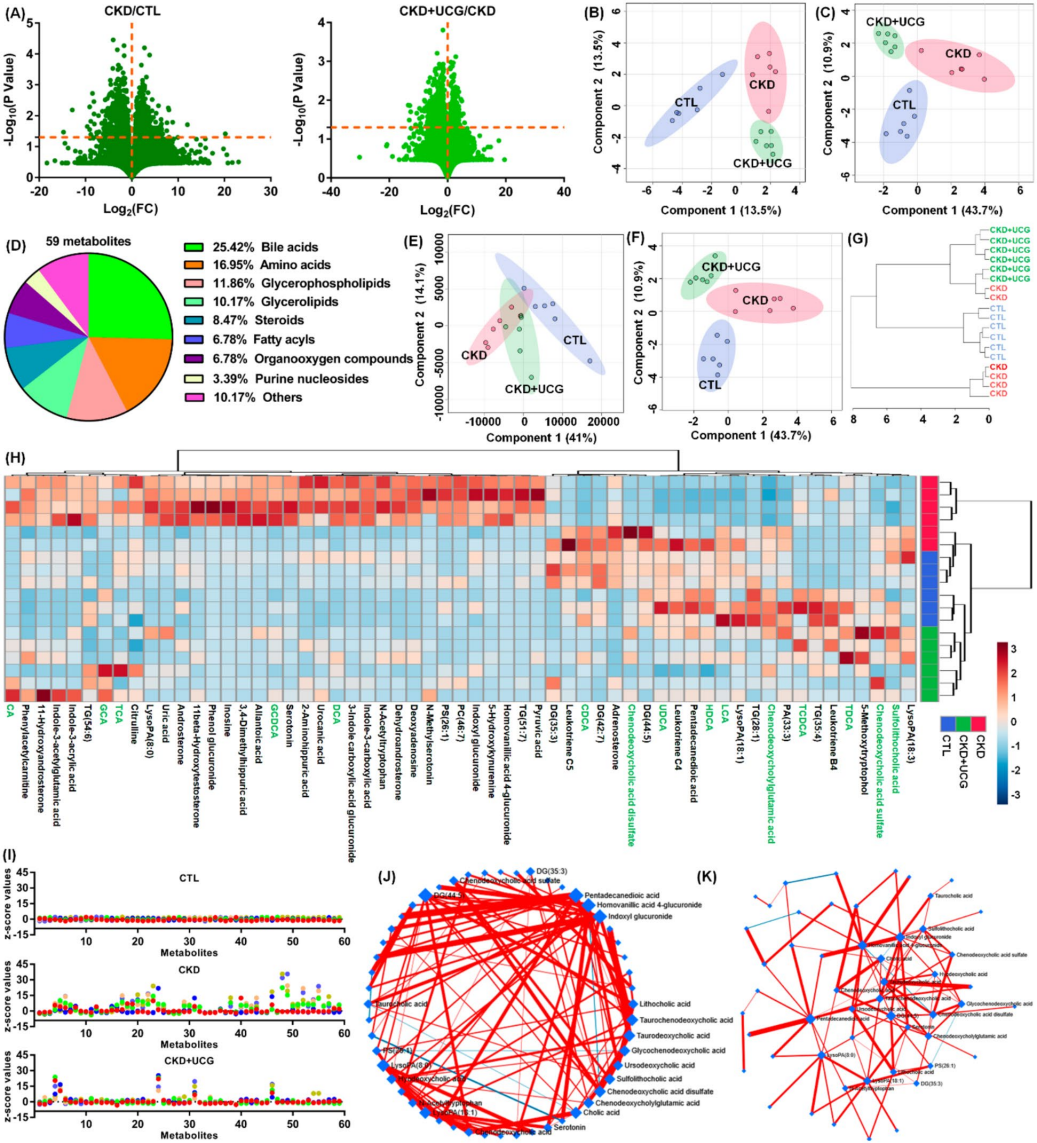
UCG antifibrotic effects were associated with altered bile acid metabolic profiles in CKD rats. **A** Geometric mean ratios of serum fragment ions in CKD/CTL and CKD + UCG/CKD comparisons in positive ion mode. **B** sPLS-DA score plots of all fragment ions in positive ion mode. **C** sPLS-DA score plots of 129 fragment ions (*P* < 0.05) identified using two-tailed unpaired Student’s t tests. **D** Pie chart showing the distribution of 59 identified metabolites. **E** PLS-DA score plots of the 59 metabolites. **F** sPLS-DA score plots of the 59 metabolites (*P* < 0.05). **G** Dendrogram of hierarchical clustering analysis of the 59 metabolites. **H** Heatmap of hierarchical clustering analysis of the 59 metabolites. **I** Z-score plot of the 59 metabolites normalized to mean values of CTL rats. **J** Circular layout of DSPC-based correlation networks of the 59 metabolites (top 20% correlations). **K** ForceAtlas layout of DSPC-based correlation networks of the 59 metabolites

To assess whether significantly altered gut microbiota were associated with changes in serum metabolite levels, serum samples from experimental rats were analyzed in positive ion mode using UPLC-HDMS. Initially, the variables were selected based on *P* < 0.05. Figure 6A shows the geometric mean ratios of the fragment ions in the serum of CKD/CTL and CKD + UCG/CKD rats. A total of 1,279 and 129 fragment ions had *P* values less than 0.05 in the CKD/CTL and CKD + UCG/CKD comparisons, respectively (Fig. 6A). sPLS-DA demonstrated that 25,474 fragment ions could clearly separate the three groups, indicating that the serum metabolic profiles were significantly altered in CKD and UCG-treated CKD rats (Fig. 6B). Similar results showed that the three groups could also be separated by 129 fragment ions with *P* < 0.05 in both CKD/CTL and CKD + UCG/CKD comparisons (Fig. 6C).

Ultimately, 59 metabolites were identified based on our previous study (Table S1) [44]. Based on their chemical structures, these 59 metabolites were further classified and were found to mainly include 15 bile acids, 10 amino acids, 7 glycerophospholipids, and 6 glycerolipids (Fig. 6D and Table S1), indicating that CKD and UCG treatment in CKD rats may have a pronounced impact on bile acid–related metabolic pathways. Both PLS-DA and sPLS-DA analyses showed that the 59 metabolites could clearly separate the three groups (Fig. 6E, F). Cluster analysis, including dendrograms and heatmaps, consistently demonstrated that 59 metabolites distinguished the three groups (Fig. 6G, H). Z-score analysis showed that most of the 59 metabolites were significantly increased in CKD rats but markedly decreased in CKD rats treated with UCG (Fig. 6I).

The circular layout of the debiased sparse partial correlation (DSPC) networks showed that bile acids, including lithocholic acid (LCA), taurochenodeoxycholic acid (TCDCA), taurodeoxycholic acid (TDCA), glycochenodeoxycholic acid (GCDCA), ursodeoxycholic acid (UDCA), sulfolithocholic acid (SCA), chenodeoxycholic acid sulfate (CDCA sulfate), chenodeoxycholylglutamic acid (Glu-CDCA), and cholic acid (CA) exhibit strong associations (Fig. 6J). Similar associations were observed in the ForceAtlas visualization of DSPC networks (Fig. 6K). These results indicate that the antifibrotic effects of UCG are associated with alterations in the bile acid–related metabolic profiles of CKD rats.

### UCG treatment reversed aberrant bile acid levels in CKD rats

**Fig. 7:**
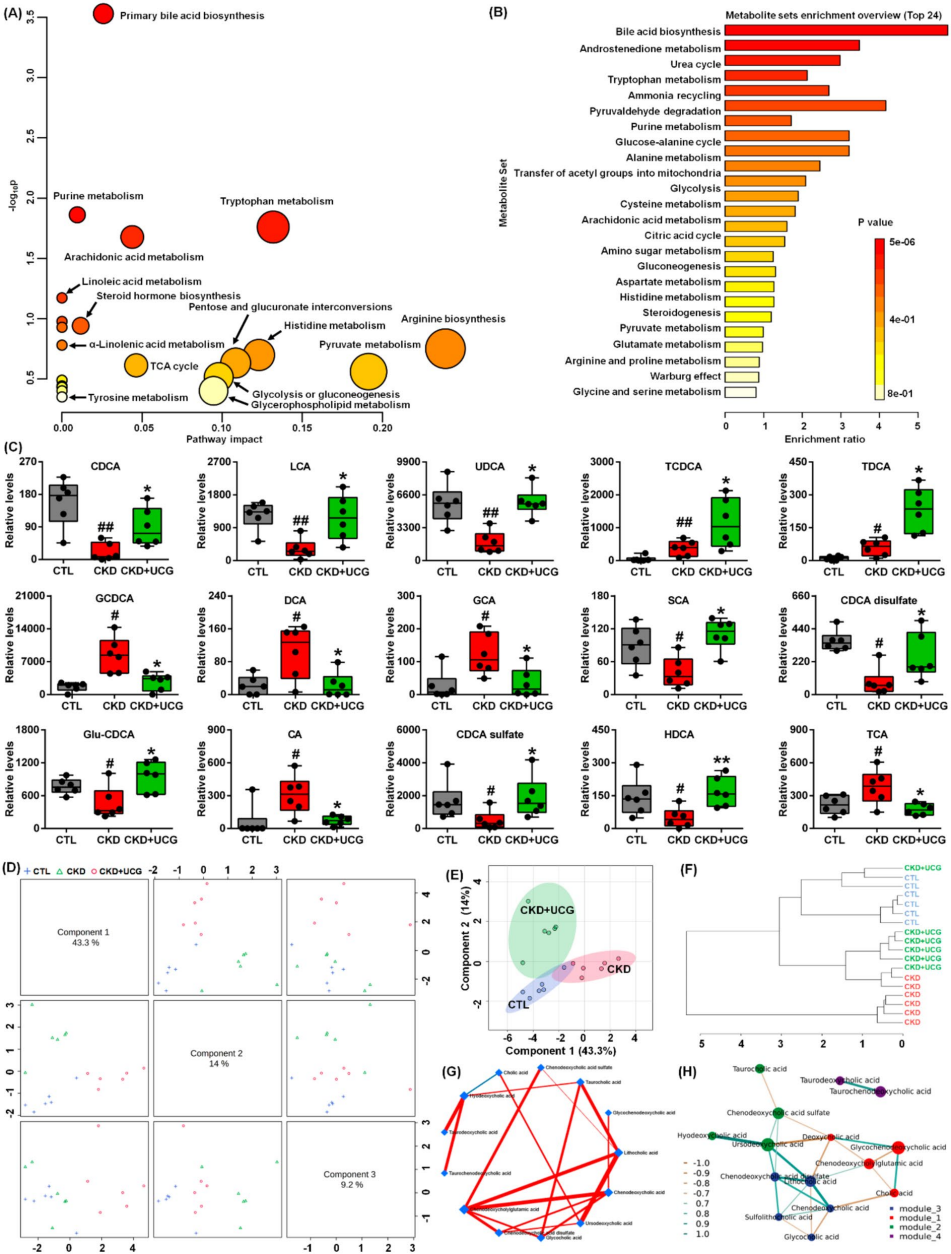
UCG treatment reversed aberrant bile acid levels in CKD rats. **A** Enrichment analysis of the 59 metabolites. **B** Metabolic pathway analysis based on the KEGG metabolic library. **C** Serum levels of 15 primary and secondary bile acids in CTL, adenine-induced CKD, and UCG-treated CKD rats. **D** sPLS-DA score plots of 15 bile acids based on different principal components. **E** sPLS-DA score plots of 15 bile acids based on two principal components. **F** Dendrogram of hierarchical clustering analysis of 15 bile acids in the three groups. **G** Circular layout of DSPC-based correlation networks of bile acids. **H** Correlation network of 15 bile acids based on Spearman correlation coefficients (*P* < 0.05). ^#^*P* < 0.05, ^##^*P* < 0.01 compared with CTL rats; *P < 0.05, **P < 0.01 compared with CKD rats

To uncover the functional roles of the 59 metabolites, the KEGG metabolic library was analyzed. The results revealed that primary bile acid biosynthesis was the most significantly altered pathway in this study (Fig. 7A and Table S2). Enrichment analysis of the 59 metabolites further confirmed this finding (Fig. 7B and Table S3). These results suggest that CKD and UCG treatment in CKD rats induce alterations in serum metabolic profiles that are closely associated with dysregulation of bile acid biosynthesis. Compared with CTL rats, adenine-induced CKD rats showed a significant increase in TCDCA, TDCA, GCDCA, deoxycholic acid (DCA), glycocholic acid (GCA), CA, and taurocholic acid (TCA), as well as a significant decrease in chenodeoxycholic acid (CDCA), LCA, UDCA, SCA, CDCA disulfate, Glu-CDCA, CDCA sulfate, and hyodeoxycholic acid (HDCA) in rat serum (Fig. 7C). However, UCG treatment reversed these aberrant bile acid changes in CKD rats (Fig. 7C), indicating that UCG treatment ameliorates bile acid dysregulation in CKD rats.

Based on distinct principal components, score plots from sparse partial least squares discriminant analysis (sPLS-DA) showed that 15 bile acids clearly separated the three groups (Fig. 7D). Similar results were also observed when two principal components were used (Fig. 7E). Moreover, the dendrogram from hierarchical clustering analysis further confirmed these findings (Fig. 7F). The circular layout of the DSPC network demonstrated that CDCA, CDCA sulfate, GCA, UDCA, and LCA exhibited strong associations (Fig. 7G). Correlation network analysis indicated that the 15 bile acids were divided into four modules, with interactions observed among three of these modules through shared metabolites (Fig. 7H). These results demonstrate that UCG treatment regulates bile acid production and metabolism in CKD rats.

### Altered bile acids as potential biomarkers for UCG treatment of CKD

**Fig. 8:**
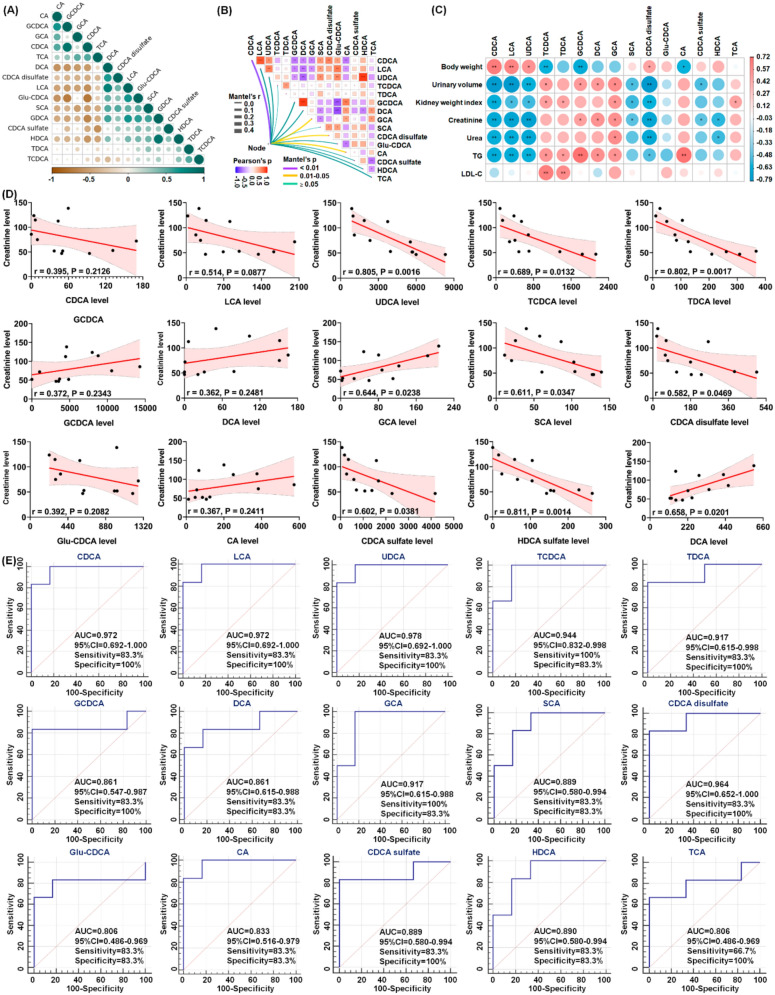
Altered bile acids as potential biomarkers for UCG treatment of CKD. **A** Correlation coefficients among 15 bile acids. Circle size indicates correlation strength; green indicates positive correlation and brown indicates negative correlation. **B** Network heatmap of 15 bile acids and nine clinical indices based on Euclidean distance, Pearson correlation coefficients, Bray–Curtis distance, and Mantel tests. **C** Correlation heatmap of 15 bile acids and nine clinical indices based on Spearman correlation coefficients (*P* < 0.05). **D** Linear correlations between serum creatinine levels and relative intensities of 15 bile acids. Red shading indicates 95% confidence bands. **E** PLS-DA–based ROC curves of 15 bile acids, with AUC, 95% CI, sensitivity, and specificity indicated

To determine whether altered bile acids could serve as potential biomarkers for UCG treatment of CKD, correlation analysis, linear regression analysis, and receiver operating characteristic (ROC) curve analysis were performed. Strong positive correlations were observed between CDCA and GCDCA, LCA and DCA, and HDCA and GDCA, whereas strong negative correlations were identified between Glu-CDCA and CA, GCDCA, and CDCA; CDCA and GDCA and HDCA; and CDCA sulfate and TCA (Fig. 8A). These findings suggest that UCG treatment regulates the bile acid metabolic network.

As shown in Fig. 8B, nine clinical parameters, including body weight, urinary volume, kidney weight index, creatinine, urea, uric acid, TC, TG, and LDL-C, were correlated with the 15 bile acids. The results indicated that CDCA exhibited the strongest correlation with the nine clinical parameters, followed by GCA, CDCA disulfate, and CA. Further analysis demonstrated that bile acids, including CDCA, LCA, UDCA, and CDCA disulfate, were positively correlated with body weight, whereas they were negatively correlated with urinary volume, kidney weight index, creatinine, urea, and TG. Moreover, HDCA were negatively correlated with the levels of creatinine and urea (Fig. 8C). Collectively, these correlations suggest that significantly altered bile acid levels are associated with adenine-induced renal injury.

Linear regression analysis between the 15 bile acids and serum creatinine levels showed that UDCA, TDCA, and HDCA had correlation coefficients greater than 0.80 (Fig. 8D), indicating that these metabolites may reflect changes in kidney function. The potential of the identified bile acids as biomarkers for UCG therapy was further evaluated using ROC curve analysis. Seven bile acids, including CDCA, LCA, UDCA, TCDCA, TDCA, GCA, and CDCA disulfate, were identified as top-ranked candidates, exhibiting an area under the ROC curve (AUC) of 0.900, along with high sensitivity and specificity (Fig. 8E). Among these candidates, UDCA exhibited the best performance in the current dataset, with an AUC of 0.978 among the 15 bile acids analyzed (Fig. 8E). The combined results of linear regression and ROC analyses indicate that UDCA, TDCA, and HDCA may serve as potential biomarkers for predicting the therapeutic effects of UCG in CKD patients.

### UCG alleviated renal fibrosis by reshaping microbial dysbiosis via bile acid metabolism in CKD rats

**Fig. 9:**
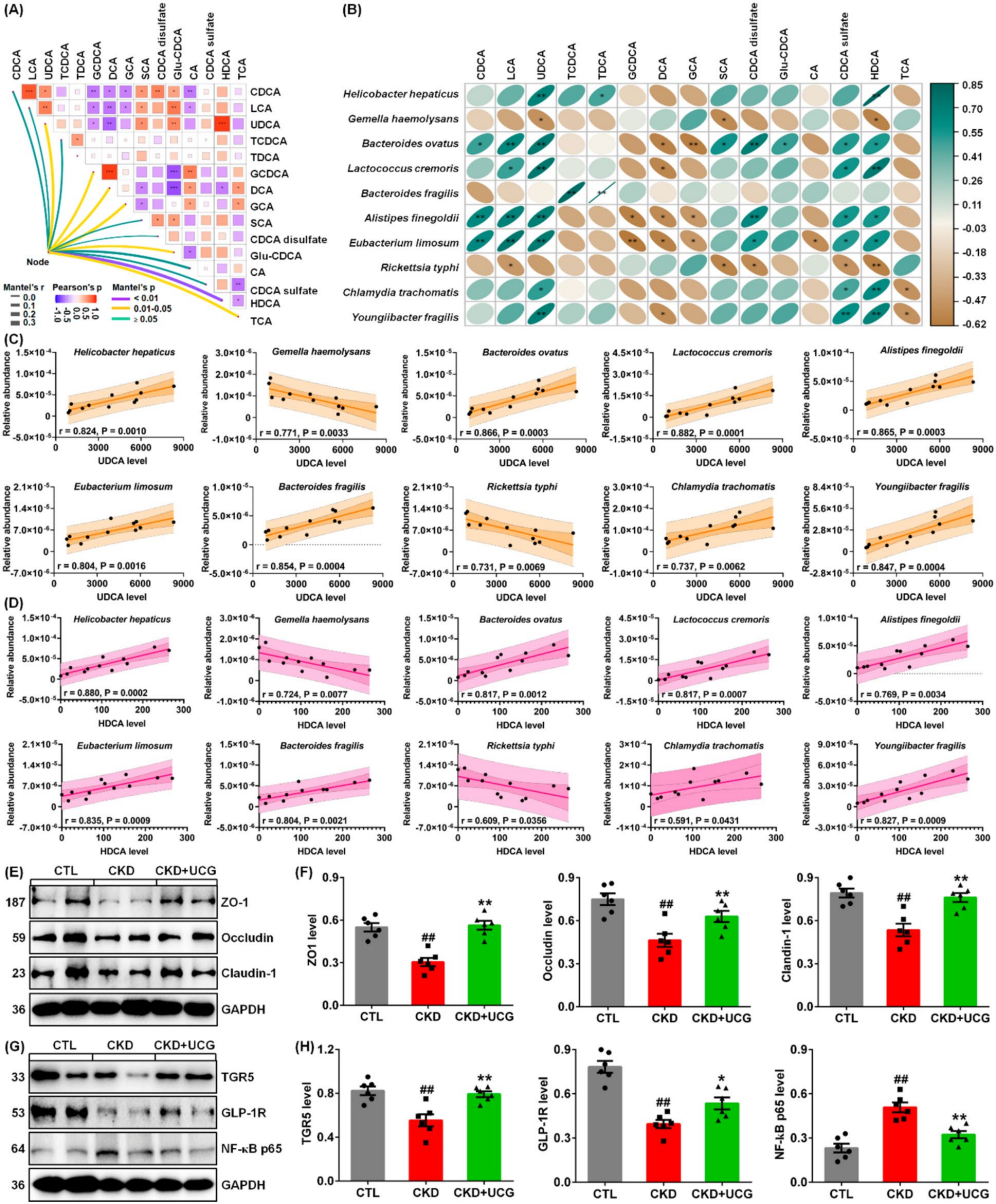
UCG treatment alleviated renal fibrosis by reshaping microbial dysbiosis via modulation of bile acid metabolism in CKD rats. **A** Network heatmap of 15 bile acids and 10 bacteria based on Euclidean distance, Pearson correlation coefficients, Bray–Curtis distance, and Mantel tests. **B** Correlation heatmap of 10 significantly altered bacteria and 15 bile acids. **C** Linear correlations between serum UDCA levels and relative abundance of 10 bacteria. Brown shading indicates 95% confidence bands; yellow shading indicates 95% prediction bands. **D** Linear correlations between serum HDCA levels and relative abundance of 10 bacteria. Red shading indicates 95% confidence bands; pink shading indicates 95% prediction bands. **E** Protein expression of ZO-1, occludin, and claudin-1 in colon tissues. **F** Quantitative analysis of ZO-1, occludin, and claudin-1 expression. **G** Protein expression of intrarenal TGR5, GLP-1R, and NF-κB p65. **H** Quantitative analysis of intrarenal TGR5, GLP-1R, and NF-κB p65. ^#^*P* < 0.05, ^##^*P* < 0.01 compared with CTL rats; *P < 0.05, **P < 0.01 compared with CKD rats

To elucidate whether the 10 fecal bacteria were correlated with the 15 serum bile acids, correlation and linear regression analyses were performed. Analysis of the interactions between the 10 bacteria and 15 bile acids showed that HDCA exhibited the strongest correlation with bacterial taxa. This was followed by UDCA, GCDCA, DCA, GCA, Gul-CDCA, and TCA (Fig. 9A).

As shown in Fig. 9B, UDCA and HDCA exhibited the strongest positive correlations with seven bacteria, including *H. hepaticus*, *B. ovatus*, *L. cremoris*, *A. finegoldii*, *E. limosum*, *C. trachomatis*, and *Y. fragilis*, as well as strong positive correlations with two additional bacteria, *G. haemolysans* and *R. typhi*. Moreover, CDCA, LCA, and CDCA sulfate showed the strongest positive correlations with four bacteria, including *B. ovatus*, *L. cremoris*, *A. finegoldii*, and *E. limosum*. In addition, GCDCA, DCA, and GCA exhibited the strongest negative correlations with three bacteria: *B. ovatus*, *A. finegoldii*, and *E. limosum*. These correlations suggest that alterations in the gut microbiota are associated with changes in bile acid profiles in CKD rats.

Linear regression analysis between the 10 bacteria and UDCA demonstrated that *B. ovatus*, *L. cremoris*, *A. finegoldii*, *E. limosum*, and *B. fragilis* showed correlation coefficients greater than 0.80 (Fig. 9C). Similarly, linear regression analysis between the 10 bacteria and HDCA showed that *H. hepaticus*, *B. ovatus*, *L. cremoris*, *E. limosum*, *B. fragilis*, and *Y. fragilis* had correlation coefficients exceeding 0.80 (Fig. 9D). Notably, both UDCA and HDCA exhibited strong linear correlations with *B. ovatus*, *L. cremoris*, *E. limosum*, and *B. fragilis*, indicating that these two metabolites may be generated by these bacteria, which is consistent with previous publications [11, 12, 48].

**Fig. 10:**
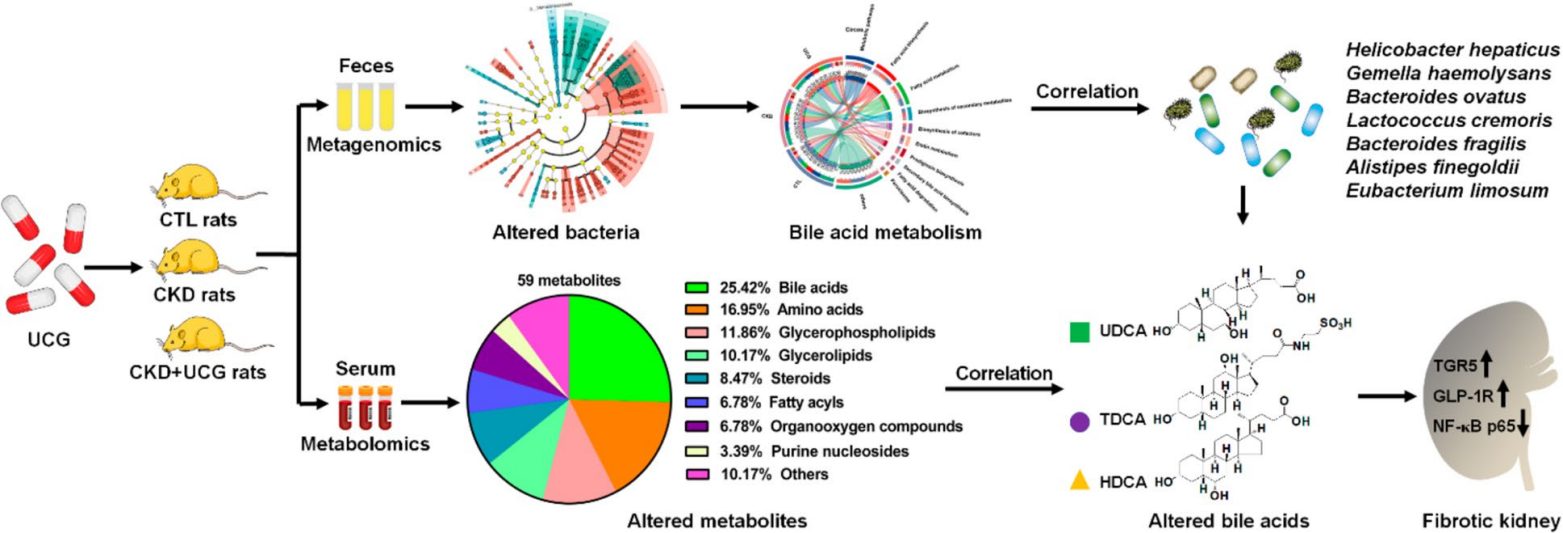
Proposed mechanism by which UCG ameliorates CKD and renal fibrosis. UCG treatment improved fecal gut microbiota dysbiosis (*H. hepaticus, G. haemolysans, B. ovatus, L. cremoris, B. fragilis, A. finegoldii, and E. limosum*) and normalized serum bile acid disorders (UDCA, TDCA, and HDCA) in adenine-induced CKD rats. UCG treatment regulates intrarenal TGR5, GLP-1R, and NF-κB p65 protein expression. Collectively, UCG may ameliorate CKD and renal fibrosis by improving TGR5, GLP-1R, and NF-κB signaling through the regulation of microbial dysbiosis–mediated bile acid metabolism

We further investigated whether UCG treatment affects the integrity of the injured intestinal epithelial barrier. The results showed that excessive adenine downregulated the expression of ZO-1, occludin, and claudin-1 in the colonic tissues of CKD rats compared to CTL rats (Fig. 9E, F). However, UCG treatment significantly upregulated the expression of these proteins in the colonic tissues of CKD rats (Fig. 9E, F). To elucidate the microbial dysbiosis–based molecular mechanisms underlying the effects of UCG treatment, we examined the protein expression levels of TGR5, GLP-1R, and NF-κB p65. The results showed that excessive adenine downregulated protein expression of TGR5, and GLP-1R as well as upregulated NF-κB p65 protein expression in the kidney tissues of CKD rats compared to CTL rats (Fig. 9G, H). In contrast, UCG treatment significantly upregulated the expression of these proteins in the kidney tissues of CKD rats (Fig. 9G, H). These findings suggest that UCG may ameliorate CKD and renal fibrosis by regulating TGR5, GLP-1R, and NF-κB p65 signaling through the regulation of microbial dysbiosis–mediated bile acid metabolism (Fig. 10).

## Discussion

Accumulating evidence has highlighted the involvement of gut microbiota dysbiosis in CKD [49–51]. In this study, we demonstrated that UCG ameliorated CKD and renal fibrosis by reshaping microbial dysbiosis. Fecal samples from adenine-induced CKD rats exhibited gut microbiota dysbiosis, and bacteria, including *H. hepaticus, G. haemolysans, B. ovatus, L. cremoris, B. fragilis, A. finegoldii*, and *E. limosum*, showed strong correlations with serum creatinine levels in CKD rats. Treatment with UCG reversed the aberrant changes observed in these bacteria. TCM has been practiced for centuries and is increasingly being recognized as a therapeutic approach for CKD and its complications [52–57]. In CKD, growing evidence indicates that gut microbiota dysbiosis can be regulated by various TCM herbs such as Rhubarb, Poria cocos, and Astragali radix, which are components of UCG [58–60]. A recent study demonstrated that neohesperidin abrogated renal fibrosis by increasing the relative abundance of *B. ovatus* [11]. In addition, the natural compound madecassoside increases the relative abundance of *B. fragilis* and attenuates renal fibrosis [12]. These findings support the hypothesis that UCG ameliorates CKD by reshaping microbial dysbiosis.

Substantial evidence suggests that microbial-derived metabolites play a critical role in linking the gut microbiota and host [13, 50]. Our second major finding showed that serum bile acids, including UDCA, TDCA, and HDCA, were strongly correlated with serum creatinine levels in CKD rats, and that UCG treatment regulated these aberrant metabolites. Increasing evidence has highlighted the dysregulation of microbial-derived bile acid metabolism in kidney diseases [61, 62]. Microbial-derived secondary bile acids (SBA) are reabsorbed and sensed by host receptors, thereby inhibiting cellular inflammation and fibrosis. An earlier publication reported significantly increased levels of bile acids and DCA in the serum of patients with chronic renal failure (CRF), and slightly increased UDCA levels and decreased levels of LCA, CA, and CDCA were observed in CRF patients compared with healthy controls [63]. Similarly, another study reported elevated serum bile acid levels in patients with CRF [64]. A previous study showed significantly increased levels of primary bile acids (PBA), including GCA, GCDCA, TCA, TCDCA, THCA, and TUDCA, but decreased SBA, including CA, CDCA, DCA, and HDCA, in ESRD patients compared with healthy controls [65]. Further analysis identified six bile acids (GCDCA, CDCA, DCA, TCDCA, GCA, and CA) as potential biomarkers for differentiating ESRD patients from healthy controls [65]. Decreased levels of CDCA, DCA, and CA are associated with dyslipidemia in ESRD patients [65]. Subgroup analysis further showed that increased levels of TCA, TCDCA, THCA, and tauroα-muricholic acid were correlated with poor prognosis in patients with ESRD [65].

Diabetic kidney disease (DKD) develops in > 40% of patients with diabetes and is a leading cause of CKD worldwide. Metabolomic analysis revealed that 50 bile acids were significantly altered in the plasma and feces but showed limited changes in urine in DKD patients [66]. Specifically, eight, eight, and three bile acids were abnormally expressed in the plasma, feces, and urine, respectively [66]. These alterations were accompanied by increased plasma conjugated/unconjugated ratios of CA, DCA, CDCA, UDCA, and HCA as well as increased fecal CA, CDCA, and LCA. Moreover, increased plasma GCDCA levels and increased fecal levels of GLCA, 7-ketodeoxycholic acid, and CDCA-3-β-D-glucuronide were correlated with eGFR, 24 h urinary protein, microalbumin, and 24 h urinary parameters that reflect DKD progression [66]. Clinical data analysis showed that higher levels of total primary bile acids, CA, CDCA, GCA, and GCDCA were associated with a lower likelihood of CKD among patients with newly diagnosed type 2 diabetes [67]. These findings suggest that higher circulating levels of unconjugated primary bile acids and their glycine conjugates are associated with lower risk of CKD in patients with type 2 diabetes [46]. In addition, higher bile acid levels were associated with lower lipid levels in patients with diabetes mellitus undergoing maintenance hemodialysis, and bile acids were identified as an independent risk factor for all-cause death in this population [68]. Moreover, a recent study showed increased serum levels of GCDCA, TCDCA, TDCA, LCA, GLCA, and TLCA in patients with autosomal dominant polycystic kidney disease [69].

Several clinical studies have examined the association between DCA and the risk of cardiovascular events, ESRD, and mortality in CKD [49–53]. The Chronic Renal Insufficiency Cohort (CRIC) study reported that DCA levels above the median were independently associated with ESRD and all-cause mortality in patients with CKD stages 2–4 [70]. Further clinical findings showed that higher serum DCA levels were independently associated with greater baseline coronary artery calcification (CAC) volume scores and lower baseline bone mineral density in patients with moderate-to-severe CKD [71]. Moreover, CKD increases serum DCA levels in mice and patients by reducing urinary DCA excretion [72]. A recent study reported that higher DCA levels were independently associated with prevalent cognitive impairment in category fluency in patients with CKD stages 2–4 [52]. In contrast, a modified Mini-Mental State Examination indicated that the association between DCA and progressive cognitive impairment was not clinically significant [73]. However, another CRIC study reported that DCA was not associated with the prevalence, incidence, or progression of CAC [74]. Mechanistic studies revealed that DCA treatment induced vascular calcification and osteogenic differentiation via endoplasmic reticulum (ER) stress–induced activation of transcription factor 4 in cultured muscle cells [72]. Treatment with farnesoid X receptor (FXR) agonists reduced serum levels of CA-derived bile acids, including DCA, and ameliorated CKD-dependent medial calcification and atherosclerotic calcification in mice [72]. Conversely, FXR deficiency and DCA treatment promote vascular calcification by increasing serum DCA levels and activating ER stress responses [72].

Several studies have highlighted the beneficial effects of CDCA on fibrosis and nephrotoxicity by suppressing inflammation and oxidative stress and by blocking the renin–angiotensin system via AT2R and ACE2 mRNA upregulation in rats [75–77]. Metabolomic profiling also showed an increase in TDCA levels during the AKI-to-CKD transition [78]. Further in vitro experiments demonstrated that TDCA activated FXR, leading to inhibition of organic anion transporter 2 expression [78]. Both in vivo and in vitro results showed that TDCA-induced downregulation of organic anion transporter 2 was reversed by the FXR inhibitor guggulsterone or by FXR knockdown [78].

Kidney transplantation (KT) is the preferred treatment option for ESRD. A clinical study showed that kidney transplant recipients (KTRs) exhibited distinct bile acid profiles compared with healthy controls [79]. Further analysis demonstrated that KTRs with severe chronic allograft dysfunction had lower levels of unconjugated bile acids and SBAs than healthy controls [79]. KTRs also showed lower SBA/PBA ratios than healthy controls, whereas conjugated/unconjugated bile acid ratios increased with allograft function deterioration. These alterations were associated with the expression of cytochrome P450 family 7 subfamily A member 1 and cytochrome P450 family 27 subfamily A member 1, which were positively correlated with eGFR [79].

Intriguingly, our third major finding revealed that both UDCA and HDCA showed high linear correlations with *B. ovatus, L. cremoris, E. limosum*, and *B. fragilis*, suggesting that these metabolites are associated with these bacterial species. UDCA, a bile acid primarily used for liver disorders, also exhibits antioxidant activity and potential protective effects in various toxicological conditions. Acute kidney injury (AKI) is a global health problem associated with a risk of progression. Recent studies have demonstrated that UDCA protects against AKI induced by renal ischemia–reperfusion injury (IRI), sepsis, cadmium, or cisplatin via multiple mechanisms, including promotion of fatty acid oxidation, improvement of mitochondrial dysfunction through regulation of aldehyde dehydrogenase 1 family member L2, activation of the nuclear factor erythroid 2-related factor 2/heme oxygenase-1 pathway, and inhibition of NF-κB signaling [48, 80, 81]. An earlier clinical analysis evaluated UDCA in hemodialysis patients with chronic hepatitis C and reported that UDCA reduced alanine aminotransferase levels in hemodialysis patients with hepatitis C virus infection [82]. A case report indicated that increasing avacopan dosage with concomitant UDCA use reduced the risk of C5a receptor inhibitor–mediated liver injury in antineutrophil cytoplasmic antibody–associated vasculitis [83]. Another study demonstrated that toxic CA and non-toxic UDCA induce adaptive ABC transporter expression in an FXR-independent manner. Multidrug resistance–associated protein (Mrp), an organic anion transporter, plays an essential role in biliary excretion of endogenous and exogenous compounds. This study suggests that intestinal and renal Mrp2 and hepatic Mrp3 induction by UDCA contribute to its therapeutic effects by promoting alternative excretory routes for bile acids and other cholephiles [84]. DKD is one of the most common and severe microvascular complications of diabetes mellitus and is increasingly being recognized as the leading cause of ESRD worldwide. Several studies have reported that UDCA ameliorates diabetes and DKD by inhibiting oxidative stress and ER stress [85–87]. In addition, UDCA has been shown to reduce nephrotoxicity by regulating NF-κB, endothelial nitric oxide synthase, and caspase-3 expression [88].

A recent study reported that renal IRI decreased UDCA levels, a metabolite of *E. limosum*, in mouse cecal content and serum [48]. In contrast, supplementation with either *E. limosum* or UDCA prevented these changes in the IRI mice. Mechanistically, UDCA directly binds and activates peroxisome proliferator-activated receptor-γ to enhance fatty acid oxidation, thereby increasing ATP production and reducing lipid accumulation in proximal tubular epithelial cells, ultimately protecting against IRI [48]. Importantly, while patients with renal IRI exhibit decreased serum UDCA levels, those with higher pre-IRI UDCA levels or higher *E. limosum* abundance develop less severe IRI [48]. Feline CKD is characterized by renal inflammation and fibrosis and mirrors the key pathophysiological features of human CKD. Cats with CKD showed altered SBA levels and decreased fecal UDCA levels compared with healthy cats [89]. CKD cats also exhibited a significant reduction in Peptacetobacter hiranonis abundance, which was positively correlated with DCA and LCA. In addition, three Oscillospirales ASVs and Roseburia ASV were correlated with fecal SBA levels in cats with CKD [89]. Previous studies have reported that streptozotocin-induced DKD mice exhibited increased 24-h urinary albumin levels and increased total fecal bile acids (especially CA and DCA), along with gut microbiome alterations [90]. DKD mice also showed increased serum total bile acids and altered bile acid profiles (including TCA, DCA, β-muricholic acid, and tauroβ-muricholic acid), which were associated with upregulated intrarenal FXR expression [91]. However, QiDiTangShen granules reshaped microbial dysbiosis and improved bile acid profiles without affecting intrarenal FXR expression in DKD mice [91]. Another recent study showed that *Oscillibacter* and UCG-005 are associated with CA, GCA, and DHCA in streptozotocin-induced DKD mice, along with increased hepatic expression of FXR, cytochrome P450 family 7 subfamily A member 1, and cytochrome P450 family 8 subfamily B member 1. In contrast, the Yiqi Wenyang Formula attenuated DKD by suppressing inflammation and modulating the gut microbiota–bile acid axis via FXR signaling in mice [92]. Moreover, a recent study reported that KTRs exhibited significantly altered abundance of *Streptococcus, Enterococcaceae*, and *Ruminococcus*, which correlated with bile acid metabolism [93]. These findings support the hypothesis that UCG ameliorates CKD by regulating bile acid metabolism.

Our fourth major finding revealed that UCG treatment regulated the intrarenal protein expression of TGR5, GLP-1R, and NF-κB p65 in CKD rats. Bile acids are among the most abundant metabolites in the gut and are essential not only for fat digestion and absorption, but also for regulating lipid, glucose, and energy metabolism [94]. The bile acid receptor TGR5, also known as G protein-coupled bile acid receptor 1, is mainly expressed in the liver, adipose tissue, and intestine and is a key mediator of bile acid–driven energy metabolism [95, 96]. A recent study showed that B. ovatus inhibits renal fibrosis by increasing gut HDCA levels by enhancing the abundance of C. scindens, which generates HDCA in mice [11]. Furthermore, HDCA increases GLP-1 expression by upregulating TGR5 and downregulating FXR expression in the gut [11]. Activation of intrarenal GLP-1R attenuates renal fibrosis [11]. Moreover, HDCA mitigated renal fibrosis by directly upregulating intrarenal TGR5 expression [11]. Another study reported a decreased abundance of *B. fragilis* in fecal samples from CKD patients and mice with unilateral ureteral obstruction (UUO) [12]. *B. fragilis* supplementation reduces lipopolysaccharide levels and attenuates UUO- and adenine-induced renal fibrosis in mice [12]. Furthermore, *B. fragilis* supplementation increased 1,5-anhydroglucitol levels that ameliorates renal fibrosis by inhibiting oxidative stress and inflammation [12]. Accumulating evidence has demonstrated that TCM formulations (e.g., Wen-Shen-Jian-Pi-Hua-Tan decoction and *Polyporus umbellatus*) and natural products (e.g., gentiopicroside and ergone) ameliorate CKD by regulating bile acid metabolism, activating TGR5, and suppressing the NF-κB pathway [97–99]. The present findings and previous studies suggest that UCG may ameliorate CKD and renal fibrosis by improving TGR5, GLP-1R, and NF-κB p65 signaling through the regulation of microbial dysbiosis–mediated bile acids.

This study has several limitations. Firstly, the results are not further validated using additional animal models of CKD. Secondly, no studies are conducted the effect of UCG on the fecal gut microbiol dysbiosis and serum metabolite disorders in CKD patients. Thirdly, the underlying molecular mechanism by which UCG regulates gut microbiol dysbiosis and serum metabolite disorder is presently unclear and requires further investigation.

## Conclusion

Our study is the first to demonstrate that UCG ameliorates CKD and renal fibrosis by reshaping microbial dysbiosis and regulating bile acid metabolism, which are associated with altered protein expression of TGR5, GLP-1R, and NF-κB p65. Intriguingly, both UDCA and HDCA showed strong linear correlations with *B. ovatus, L. cremoris, E. limosum*, and *B. fragilis* in CKD rats. These gut bacteria and their associated metabolites, including UDCA and HDCA, may serve as biomarkers to predict the therapeutic efficacy of UCG in CKD.

## Supplementary Information

Additional file1 (DOCX 39 KB)

## Data Availability

The datasets supporting this study’s conclusions are accessible through the corresponding author upon a reasonable request.

## Abbreviations

B. fragilis: *Bacteroides fragilis*
B. ovatus: *Bacteroides ovatus*
C. scindens: *Clostridium scindens*
CA: Cholic acid
CAC: Coronary artery calcification
CDCA: Chenodeoxycholic acid
CDCA sulfate: Chenodeoxycholic acid sulfate
CKD: Chronic kidney disease
CRIC: Chronic renal insufficiency cohort
CTL: Control
DCA: Deoxycholic acid
DSPC: Debiased sparse partial correlation
E. lenta: *Eggerthella lenta*
ECM: Extracellular matrix
ER: Endoplasmic reticulum
ESRD: End-stage renal disease
F. nucleatum: *Fusobacterium nucleatum*
F. prausnitzii: *Faecalibacterium prausnitzii*
FDR: False discovery rate
FXR: Farnesoid X receptor
G. haemolysans: *Gemella haemolysans*
GCDCA: Glycochenodeoxycholic acid
GLP-1: Glucagon-like peptide-1
GLP-1R: Glucagon-like peptide-1 receptor
glu-CDCA: Chenodeoxycholylglutamic acid
H&E: Hematoxylin–eosin
H. hepaticus: *Helicobacter hepaticus*
HDCA: Hyodeoxycholic acid
IAld: Indole-3-aldehyde
IRI: Ischemia–reperfusion injury
KTR: Kidney transplant recipients
L. cremoris: *Lactococcus cremoris*
L. johnsonii: *Lactobacillus johnsonii*
LCA: Lithocholic acid
LDL-C: Low-density lipoprotein-cholesterol
Mrp: Multidrug resistance associated protein
NF-κB: Nuclear factor kappa B
PBA: Primary bile acid
PCA: Principal component analysis
PCoA: Principal coordinates analysis
PLS-DA: Partial least squares-discriminant analysis
ROC: Receiver operating characteristic
SBA: Secondary bile acids
SCA: Sulfolithocholic acid
sPLS-DA: Sparse partial least squares-discriminant analysis
TC: Total cholesterol
TCA: Taurocholic acid
TCDCA: Taurochenodeoxycholic acid
TCM: Traditional Chinese medicine
TDCA: Taurodeoxycholic acid
TG: Triglycerides
TGR5: Takeda G protein-coupled receptor 5
UCG: Uremic clearance granule
UDCA: Ursodeoxycholic acid
ZO-1: Zonula occludens-1
α-SMA: Alpha-smooth muscle actin
