# Supplementary material for: Ureic clearance granule ameliorates chronic kidney disease by reshaping microbial dysbiosis via modulating bile acid metabolism
Source: Chin Med. 2026 May 29;21:155. doi: 10.1186/s13020-026-01423-y (PMC13220541; doi:10.1186/s13020-026-01423-y)
Supplement: Supplementary file 1 — Additional file1 (DOCX 39 KB) [file 13020_2026_1423_MOESM1_ESM.docx]

**Supplementary material**

**Ureic clearance granule ameliorates chronic kidney disease by reshaping microbial dysbiosis via modulating bile acid metabolism**

Table S1. Identified serum metabolites, FC, P values and FDR among the CTL, CKD and CKD+UCG group

| Metabolites | FC^a^ | P^b^ | FDR^c^ | FC^a^ | P^b^ | FDR^c^ | Class |
| --- | --- | --- | --- | --- | --- | --- | --- |
| CDCA | 0.13 | 6.40E-04 | 3.78E-02 | 4.01 | 4.58E-02 | 8.46E-02 | BA |
| LCA | 0.26 | 6.55E-04 | 1.93E-02 | 3.69 | 4.45E-02 | 1.00E-01 | BA |
| UDCA | 0.34 | 3.25E-03 | 4.80E-02 | 2.99 | 1.47E-02 | 9.50E-02 | BA |
| TCDCA | 7.81 | 8.85E-03 | 6.53E-02 | 3.03 | 4.03E-02 | 7.22E-02 | BA |
| TDCA | 5.78 | 1.00E-02 | 5.39E-02 | 3.91 | 1.71E-02 | 8.23E-02 | BA |
| GCDCA | 5.08 | 1.34E-02 | 5.27E-02 | 0.32 | 3.51E-02 | 7.66E-02 | BA |
| DCA | 4.68 | 1.44E-02 | 4.99E-02 | 0.21 | 1.77E-02 | 7.55E-02 | BA |
| GCA | 4.67 | 2.32E-02 | 5.26E-02 | 0.28 | 3.37E-02 | 6.97E-02 | BA |
| SCA | 0.45 | 2.63E-02 | 5.54E-02 | 2.76 | 1.52E-02 | 6.50E-02 | BA |
| CDCA disulfate | 0.21 | 3.94E-02 | 5.95E-02 | 3.03 | 4.44E-02 | 6.20E-02 | BA |
| Glu-CDCA | 0.60 | 4.34E-02 | 5.22E-02 | 2.07 | 1.41E-02 | 6.84E-02 | BA |
| CA | 5.71 | 4.40E-02 | 5.00E-02 | 0.21 | 3.41E-02 | 6.50E-02 | BA |
| CDCA sulfate | 0.29 | 4.47E-02 | 4.98E-02 | 3.86 | 3.21E-02 | 6.24E-02 | BA |
| HDCA | 0.34 | 3.57E-02 | 5.69E-02 | 3.48 | 4.83E-03 | 4.81E-02 | BA |
| TCA | 1.76 | 4.85E-02 | 5.11E-02 | 0.46 | 1.28E-02 | 5.95E-02 | BA |
| Citrulline | 1.71 | 1.05E-02 | 5.14E-02 | 0.35 | 1.43E-03 | 5.59E-02 | AA |
| Serotonin | 18.9 | 1.34E-02 | 4.94E-02 | 0.10 | 1.74E-02 | 5.43E-02 | AA |
| Indole-3-acetylglutamic acid | 3.40 | 1.87E-02 | 5.26E-02 | 0.16 | 9.45E-03 | 5.21E-02 | AA |
| Indole-3-acrylic acid | 4.55 | 2.60E-02 | 5.69E-02 | 0.20 | 2.55E-02 | 4.98E-02 | AA |
| 5-Hydroxykynurenine | 6.03 | 2.66E-02 | 5.41E-02 | 0.14 | 2.24E-02 | 5.21E-02 | AA |
| N-Acetyltryptophan | 3.77 | 3.47E-02 | 6.02E-02 | 0.28 | 4.11E-02 | 5.04E-02 | AA |
| 3,4-Dimethylhippuric acid | 6.51 | 3.94E-02 | 5.81E-02 | 0.08 | 2.71E-02 | 4.89E-02 | AA |
| N-Methylserotonin | 6.90 | 3.98E-02 | 5.59E-02 | 0.03 | 2.33E-02 | 4.74E-02 | AA |
| 5-Methoxytryptophol | 9.20 | 4.90E-02 | 4.98E-02 | 4.77 | 4.40E-02 | 4.75E-02 | AA |
| 2-Aminohippuric acid | 2.95 | 4.97E-02 | 4.97E-02 | 0.13 | 1.68E-02 | 4.57E-02 | AA |
| LysoPA(18:3) | 0.10 | 8.97E-03 | 5.88E-02 | 6.96 | 4.33E-02 | 4.39E-02 | GP |
| PA(33:3) | 0.36 | 9.80E-03 | 5.78E-02 | 3.42 | 3.84E-02 | 5.08E-02 | GP |
| LysoPA(18:1) | 0.36 | 1.54E-02 | 5.06E-02 | 3.26 | 3.33E-02 | 4.98E-02 | GP |
| LysoPA(8:0) | 3.94 | 1.93E-02 | 4.96E-02 | 0.24 | 3.50E-02 | 4.92E-02 | GP |
| PS(26:1) | 5.71 | 2.01E-02 | 4.93E-02 | 0.12 | 1.32E-02 | 4.88E-02 | GP |
| TG(35:4) | 3.37 | 2.79E-02 | 5.48E-02 | 3.20 | 1.05E-02 | 4.72E-02 | GP |
| PC(46:7) | 4.16 | 4.88E-02 | 5.05E-02 | 0.03 | 9.92E-03 | 4.60E-02 | GP |
| DG(42:7) | 0.09 | 9.23E-04 | 1.82E-02 | 4.36 | 8.95E-03 | 4.71E-02 | GL |
| TG(28:1) | 0.10 | 6.79E-03 | 8.01E-02 | 19.5 | 2.28E-02 | 4.84E-02 | GL |
| DG(44:5) | 0.06 | 6.91E-03 | 6.79E-02 | 8.85 | 3.12E-02 | 4.90E-02 | GL |
| TG(54:6) | 2.36 | 1.64E-02 | 4.83E-02 | 0.31 | 7.78E-03 | 5.26E-02 | GL |
| DG(35:3) | 0.33 | 2.94E-02 | 5.43E-02 | 0.18 | 3.85E-02 | 5.25E-02 | GL |
| TG(51:7) | 6.54 | 4.11E-02 | 5.38E-02 | 0.00 | 1.86E-02 | 5.20E-02 | GL |
| Adrenosterone | 0.43 | 1.09E-02 | 4.93E-02 | 0.05 | 3.40E-03 | 5.16E-02 | SSD |
| 11-Hydroxyandrosterone | 4.19 | 3.41E-02 | 6.10E-02 | 0.19 | 2.82E-02 | 5.10E-02 | SSD |
| Dehydroandrosterone | 3.66 | 3.91E-02 | 6.08E-02 | 0.21 | 3.49E-02 | 5.03E-02 | SSD |
| Androsterone | 2.41 | 4.08E-02 | 5.47E-02 | 0.32 | 2.40E-02 | 5.02E-02 | SSD |
| 11beta-Hydroxytestosterone | 4.73 | 4.59E-02 | 5.02E-02 | 0.13 | 3.26E-02 | 4.92E-02 | SSD |
| Leukotriene C5 | 0.10 | 1.30E-02 | 5.47E-02 | 2.70 | 4.21E-02 | 4.82E-02 | FA |
| Leukotriene C4 | 0.31 | 1.92E-02 | 5.15E-02 | 3.06 | 6.97E-03 | 4.93E-02 | FA |
| Leukotriene B4 | 2.32 | 3.96E-02 | 5.70E-02 | 2.27 | 2.40E-02 | 4.89E-02 | FA |
| Pentadecanedioic acid | 0.46 | 4.78E-02 | 5.13E-02 | 3.06 | 4.89E-03 | 4.89E-02 | FA |
| HAG | 35.5 | 1.60E-02 | 4.98E-02 | 0.02 | 1.50E-02 | 4.82E-02 | OC |
| Phenol glucuronide | 34.2 | 4.34E-02 | 5.12E-02 | 0.03 | 4.43E-02 | 4.73E-02 | OC |
| Indoxyl glucuronide | 2.98 | 2.94E-02 | 5.60E-02 | 0.28 | 1.85E-02 | 4.85E-02 | OC |
| 3-ICAG | 4.39 | 4.17E-02 | 5.35E-02 | 0.06 | 1.42E-02 | 4.78E-02 | OC |
| Inosine | 8.69 | 3.48E-02 | 5.86E-02 | 0.04 | 2.41E-02 | 4.75E-02 | PN |
| Deoxyadenosine | 4.29 | 4.05E-02 | 5.56E-02 | 0.19 | 3.81E-02 | 4.78E-02 | PN |
| Uric acid | 7.71 | 8.53E-03 | 7.19E-02 | 0.22 | 4.51E-02 | 4.82E-02 | Others |
| Allantoic acid | 16.2 | 2.26E-02 | 5.32E-02 | 0.13 | 4.05E-02 | 4.75E-02 | Others |
| Indole-3-carboxylic acid | 3.46 | 3.56E-02 | 5.83E-02 | 0.15 | 1.37E-02 | 4.67E-02 | Others |
| Pyruvic acid | 7.24 | 4.21E-02 | 5.29E-02 | 0.11 | 3.73E-02 | 4.60E-02 | Others |
| Phenylacetylcarnitine | 2.02 | 4.24E-02 | 5.21E-02 | 0.36 | 3.68E-02 | 4.58E-02 | Others |
| Urocanic acid | 2.87 | 4.40E-02 | 5.09E-02 | 0.22 | 1.86E-02 | 4.58E-02 | Others |

^a^ FC was calculated based on mean ratios for CKD vs CTL, CKD+UCG/CKD. FC with a value >1 indicates a higher intensity of the metabolites in CKD rats compared with CTL rats or CKD+UCG rats compared to CKD rats, whereas a FC value <1 indicates a lower intensity of the metabolites in CKD rats compared with CTL rats or CKD+UCG rats compared to CKD rats. ^b^ P values are calculated from a one-way ANOVA. ^c^ FDR value was obtained from the adjusted p value using the Benjamini Hochberg method. Abbreviation: 3-ICAG, 3-Indole carboxylic acid glucuronide; HAG, Homovanillic acid 4-glucuronide

Table S2. Top metabolic pathways of 59 metabolites based on low p values or high impact

| Pathway Name | Total | Hits | p | -log(p) | Holm p | FDR | Impact |
| --- | --- | --- | --- | --- | --- | --- | --- |
| Primary bile acid biosynthesis | 5 | 46 | 0.000 | 3.531 | 0.024 | 0.024 | 0.026 |
| Purine metabolism | 4 | 70 | 0.014 | 1.862 | 1 | 0.420 | 0.010 |
| Tryptophan metabolism | 3 | 41 | 0.017 | 1.760 | 1 | 0.420 | 0.132 |
| Arachidonic acid metabolism | 3 | 44 | 0.021 | 1.677 | 1 | 0.420 | 0.044 |
| Linoleic acid metabolism | 1 | 5 | 0.067 | 1.172 | 1 | 1 | 0 |
| Taurine and hypotaurine metabolism | 1 | 8 | 0.106 | 0.976 | 1 | 1 | 0 |
| Steroid hormone biosynthesis | 3 | 87 | 0.115 | 0.940 | 1 | 1 | 0.012 |
| Ascorbate and aldarate metabolism | 1 | 9 | 0.118 | 0.928 | 1 | 1 | 0 |
| α-Linolenic acid metabolism | 1 | 13 | 0.166 | 0.780 | 1 | 1 | 0 |
| Arginine biosynthesis | 1 | 14 | 0.178 | 0.750 | 1 | 1 | 0.239 |
| Histidine metabolism | 1 | 16 | 0.200 | 0.698 | 1 | 1 | 0.123 |
| Pentose and glucuronate interconversions | 1 | 19 | 0.233 | 0.632 | 1 | 1 | 0.108 |
| TCA cycle | 1 | 20 | 0.244 | 0.612 | 1 | 1 | 0.046 |
| Pyruvate metabolism | 1 | 23 | 0.276 | 0.560 | 1 | 1 | 0.191 |
| Glycolysis or gluconeogenesis | 1 | 26 | 0.306 | 0.515 | 1 | 1 | 0.098 |
| Alanine, aspartate and glutamate metabolism | 1 | 28 | 0.325 | 0.488 | 1 | 1 | 0 |
| Lipoic acid metabolism | 1 | 28 | 0.325 | 0.488 | 1 | 1 | 0 |
| Glyoxylate and dicarboxylate metabolism | 1 | 32 | 0.362 | 0.441 | 1 | 1 | 0 |
| Glycine, serine and threonine metabolism | 1 | 33 | 0.371 | 0.430 | 1 | 1 | 0 |
| Cysteine and methionine metabolism | 1 | 33 | 0.371 | 0.430 | 1 | 1 | 0 |
| Arginine and proline metabolism | 1 | 36 | 0.397 | 0.401 | 1 | 1 | 0 |
| Glycerophospholipid metabolism | 1 | 36 | 0.397 | 0.401 | 1 | 1 | 0.095 |
| Tyrosine metabolism | 1 | 42 | 0.447 | 0.350 | 1 | 1 | 0 |

Table S3. Enrichment analysis of 59 metabolites based on low p values and enrichment ratio

| Total | Hits | Expect | | P value | Holm P | FDR | Details |
| --- | --- | --- | --- | --- | --- | --- | --- |
| Bile acid biosynthesis | 65 | | 9 | 1.49 | 4.6E-06 | 4.5E-04 | 4.5E-04 |
| Androstenedione metabolism | 24 | | 2 | 0.551 | 0.102 | 1 | 1 |
| Urea cycle | 28 | | 2 | 0.643 | 0.133 | 1 | 1 |
| Tryptophan metabolism | 59 | | 3 | 1.35 | 0.149 | 1 | 1 |
| Ammonia recycling | 31 | | 2 | 0.712 | 0.157 | 1 | 1 |
| Pyruvaldehyde degradation | 10 | | 1 | 0.23 | 0.208 | 1 | 1 |
| Purine metabolism | 73 | | 3 | 1.68 | 0.232 | 1 | 1 |
| Glucose-alanine cycle | 13 | | 1 | 0.298 | 0.262 | 1 | 1 |
| Alanine metabolism | 17 | | 1 | 0.39 | 0.328 | 1 | 1 |
| Transfer of acetyl groups into mitochondria | 22 | | 1 | 0.505 | 0.403 | 1 | 1 |
| Glycolysis | 23 | | 1 | 0.528 | 0.417 | 1 | 1 |
| Cysteine metabolism | 26 | | 1 | 0.597 | 0.457 | 1 | 1 |
| Arachidonic acid metabolism | 67 | | 2 | 1.54 | 0.463 | 1 | 1 |
| Citric acid cycle | 32 | | 1 | 0.735 | 0.53 | 1 | 1 |
| Amino sugar metabolism | 33 | | 1 | 0.757 | 0.541 | 1 | 1 |
| Gluconeogenesis | 33 | | 1 | 0.757 | 0.541 | 1 | 1 |
| Aspartate metabolism | 35 | | 1 | 0.803 | 0.563 | 1 | 1 |
| Histidine metabolism | 42 | | 1 | 0.964 | 0.631 | 1 | 1 |
| Steroidogenesis | 43 | | 1 | 0.987 | 0.64 | 1 | 1 |
| Pyruvate metabolism | 47 | | 1 | 1.08 | 0.673 | 1 | 1 |
| Glutamate metabolism | 48 | | 1 | 1.1 | 0.681 | 1 | 1 |
| Arginine and proline metabolism | 52 | | 1 | 1.19 | 0.711 | 1 | 1 |
| Warburg effect | 57 | | 1 | 1.31 | 0.744 | 1 | 1 |
| Glycine and serine metabolism | 59 | | 1 | 1.35 | 0.756 | 1 | 1 |
